# *Hotair* Is Dispensible for Mouse Development

**DOI:** 10.1371/journal.pgen.1006232

**Published:** 2016-12-15

**Authors:** Ana Rita Amândio, Anamaria Necsulea, Elisabeth Joye, Bénédicte Mascrez, Denis Duboule

**Affiliations:** 1 School of Life Sciences, Ecole Polytechnique Fédérale de Lausanne (EPFL), Lausanne, Switzerland; 2 Department of Genetics and Evolution, University of Geneva, Geneva, Switzerland; Stanford University School of Medicine, UNITED STATES

## Abstract

Despite the crucial importance of *Hox* genes functions during animal development, the mechanisms that control their transcription in time and space are not yet fully understood. In this context, it was proposed that *Hotair*, a lncRNA transcribed from within the *HoxC* cluster regulates *Hoxd* gene expression in *trans*, through the targeting of Polycomb and consecutive transcriptional repression. This activity was recently supported by the skeletal phenotype of mice lacking *Hotair* function. However, other loss of function alleles at this locus did not elicit the same effects. Here, we re-analyze the molecular and phenotypic consequences of deleting the *Hotair* locus *in vivo*. In contrast with previous findings, we show that deleting *Hotair* has no detectable effect on *Hoxd* genes expression *in vivo*. In addition, we were unable to observe any significant morphological alteration in mice lacking the *Hotair* transcript. However, we find a subtle impact of deleting the *Hotair* locus upon the expression of the neighboring *Hoxc11* and *Hoxc12* genes *in cis*. Our results do not support any substantial role for *Hotair* during mammalian development *in vivo*. Instead, they argue in favor of a DNA-dependent effect of the *Hotair* deletion upon the transcriptional landscape *in cis*.

## Author Summary

During mammalian embryonic development, *Hox* genes must be tightly regulated. It was proposed earlier that part of this regulation relies upon *Hotair*, a long non-coding RNA that recruits repressive protein complexes onto the *HoxD* gene cluster to keep these genes silent before they become activated. A genetic deletion of *Hotair* in mice induced homeotic transformations, thus supporting this hypothesis. However, other alleles involving this locus gave controversial results and hence we re-assessed the effect of the full deletion of *Hotair in vivo*. In our genetic background and using our analytical conditions, we could not confirm the reported morphological alterations, nor could we detect any mis-regulation of *Hoxd* genes in those fetal tissues where *Hotair* is detected in control animals. However, the genomic deletion induces the mis-regulation in-*cis* of the neighboring *Hoxc11* and *Hoxc12* genes, a side-effect which may underlie a weakly penetrant alteration observed in the shape of some tail vertebrae.

## Introduction

*Hox* genes encode transcription factors with crucial roles in the specification of regional identities along the body axes during development. Mutations affecting specific *Hox* genes typically lead to homeotic transformations, whereby a particular body part is transformed into the identity of another one [1–4]. In mammals, following the two rounds of genome duplication that occurred at the basis of the vertebrate lineage (see [5]), four distinct clusters of *Hox* genes are found (*HoxA* to *HoxD*) (ref. in [6]). During development, *Hox* genes are transcriptionally activated in a precise temporal and spatial sequence, which follows their chromosomal order [7,8]. These collinear patterns of transcription are regulated at multiple levels and studies focusing on the *HoxA*, *HoxB* and *HoxD* loci have revealed the importance of intricate combinations of local and long-range *cis*-regulatory elements. Also, studies using micro-dissected embryonic material have shown that the transcriptional activation of these genes, in different ontogenetic contexts, is accompanied by major changes in both the epigenetic modifications of the surrounding chromatin and its 3D spatial organization [9–11].

Long non-coding RNAs (lncRNAs) have been proposed to represent yet another layer of regulatory control at these important developmental loci (e.g. [12–15]). Increasing evidence indeed suggests that lncRNAs can act as regulators of gene expression, for example by interacting with transcription factors and chromatin modifiers to modulate transcription during development [16]. Several lncRNAs associated with the mammalian *Hox* clusters have been identified, amongst which *Hotair* (*Hox* transcript antisense intergenic RNA), a lncRNA transcribed from the intergenic region between *Hoxc11* and *Hoxc12* within the *HoxC* cluster and the founding member of this new class of RNAs. *Hotair* was proposed to help repress some 5’-located (posterior) *Hoxd* genes in *trans*, through its association with chromatin modification complexes such as PRC2, LSD1 and CoREST/REST [13,17]. Accordingly, *Hotair* would recruit or enrich this part of the *HoxD* cluster with *Polycomb* (*Pc*) complex, thus contributing to its repressed state before transcription starts. This proposal was substantiated by the knockdown of *Hotair* in human fibroblasts, which led to a decreased binding of *Pc* repressive complexes in the *HoxD* cluster and to a concurrent increase in *Hoxd* genes expression [13].

**Fig 1:**
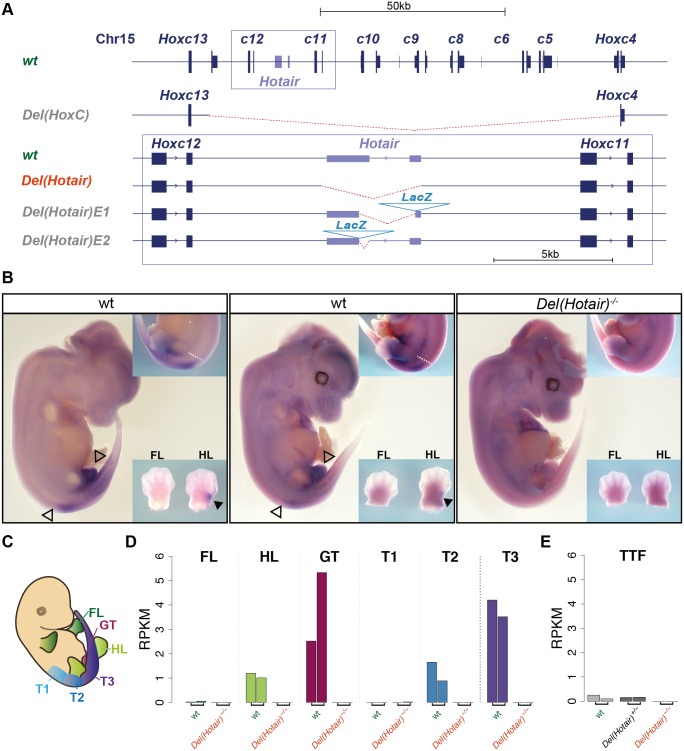
*Hotair* expression *in vivo*. (A) Schematic representation of the wild type *Hotair* locus and the various *Hotair* deletion alleles. The deleted DNA is in red. The *HoxC* allele is from [18] and the shorter deletions in the box from [20] and [21]. (B) Whole mount in situ hybridization (WISH) of *Hotair* RNAs on E12.5 wild type CD1 (left) and CBA/C57/B6 (center) control embryos and of a *Del(Hotair)*^*-/-*^ mouse embryo (right, n = 3). No signal was detected in *Del(Hotair)*^*-/-*^ embryos, demonstrating the specificity of the probe. *Hotair* is expressed with a posterior restriction (white dashed line), resembling the transcript distribution of either a *Hox11* or a *Hox12* gene. Black arrowheads indicate expression domain of *Hotair* in the hindlimbs, hollow arrowheads indicate the limit of *Hotair* expression in the trunk and in the genital tubercle. The common artifact signal in the cerebral vesicles results from incomplete opening of these vesicles and subsequent probe trapping. (C) Schematic representation of the dissection patterns for RNA-seq. These dissections involved forelimbs (FL, dark green), hindlimbs (HL, green) and the genital tubercle (GT, magenta), as well as three trunk sections corresponding to the lumbar/sacral (T1, light blue); sacro/caudal (T2, blue) and caudal (T3, purple) regions. (D) Quantification of *Hotair* expression by RNA-seq (normalized RPKM values). (E) Quantification of *Hotair* expression (normalized RPKM values) in tail tip fibroblasts (TTF), using data from [20].

This important function for a lncRNA in cultured human fibroblasts was however not supported by the analysis of a mouse line carrying a targeted deletion of the entire *HoxC* cluster [18], i.e. including the mouse *Hotair* lncRNA. This deletion showed little effect *in vivo*, with no alteration of *Hoxd* genes expression. Also, the presence and enrichment of H3K27me3 repressive chromatin marks at the *HoxD* locus was not dramatically modified [19]. This lack of effect was tentatively explained by the concomitant in-*cis* deletion of all *Hoxc* genes, which may have masked or compensated a potential alteration caused by the absence of *Hotair* alone [20]. To alleviate this problem, three alleles were recently produced where the *Hotair* transcript was specifically targeted (Fig 1). The first allele is a targeted deletion of the two major exons of *Hotair*. Mice carrying this deletion were reported to display a malformation of the wrist and homeotic transformations of the spine, either from six lumbar vertebrae (L6) to a L5 vertebral formula, or within the post-sacral region [20]. These phenotypes were associated with a de-repression of *Hoxd* genes and of a set of imprinted genes by modulation of their chromatin state [20]. These effects were scored in the absence of any change in the transcription of the neighboring *Hoxc11* and *Hoxc12* genes, supporting a function of *Hotair* in *trans* [20].

Two additional *Hotair* deletion mutant alleles combined with *LacZ* reporter knock-in were recently reported by Lai and colleagues [21]. The first allele deleted nearly the entire *Hotair* sequence and the second one comprised a smaller deletion starting in the second exon [21]. In both cases, while a subtle alteration of the 4th caudal vertebra was scored, the wrist and the spine appeared normally formed, without any sign of the lumbar homeotic transformation and wrist alterations previously reported for the deletion of both exons [20]. Due to our long-lasting interest in the transcriptional regulation of *Hoxd* genes during development (e.g. [22]), we addressed these apparently conflicting results by re-assessing the effects of deleting the *Hotair* lncRNA during early mouse development, using the largest deletion allele previously described [20]. In agreement with earlier and more recent results [19,21], we find that the deletion of *Hotair* has no substantial effect, neither on wrist morphology, nor on the vertebral formula at the lumbo-sacral level. In addition, transcriptome analyses reveal that the absence of *Hotair* does not impact upon *Hoxd* genes expression in *trans*, in any of the embryonic tissues analyzed. In contrast, we observe subtle yet significant changes in the expression of the neighboring *Hoxc11* and *Hoxc12* genes in the mutant mice, indicating an in-*cis* impact of modifying the genomic locus. Taken together, our results strongly suggest that the *Hotair* lncRNA has little effect–if any- on mouse embryonic development.

## Results

### *Hotair* expression *in vivo*

We extended the analysis of a mouse strain that includes a deletion of the two major *Hotair* exons (Fig 1A)[20]. Even though we concluded that this mutation is primarily an allele of the *HoxC* cluster (see below) and hence that it should be referred to as *HoxC*^*Del(Hotair*)^, we shall refer to it as *Del(Hotair)* throughout this study for sake of simplicity. We first confirmed the expression pattern of *Hotair in vivo* by whole mount *in situ* hybridization (WISH) using wild type mice of two distinct genetic backgrounds (CD1 and CBA/C57/B6) as well as *Del(Hotair)*^*-/-*^ mouse embryos at embryonic day 12.5 (E12.5)(Fig 1B). Staining of *Del(Hotair)*^*-/-*^ embryos confirmed the specificity of the *Hotair* probe as no signal was detected in these embryos (Fig 1B). In contrast, wild type embryos of both genetic backgrounds showed the presence of *Hotair* transcripts in the genital tubercle, the proximal part of the hindlimbs and in the caudal part of the embryo (Fig 1B), confirming previously published data [19] and consistent with the *LacZ* staining reported for the *Hotair* knocked-in allele [21]. In both cases, staining was observed just posterior to the lumbar region and was not scored in developing forelimbs [21].

We quantified the expression levels of *Hotair* with high coverage RNA-sequencing (RNA-seq) (Materials and Methods, S1 Table). Based on the spatial expression pattern of *Hotair* as determined with WISH and on the skeletal phenotypes reported in mice by Li et al. [20], we micro-dissected both *Del(Hotair)*^*-/-*^ and wild type E12.5 embryos into six distinct parts for comparative RNA-seq analyses (Fig 1C). We thus separately collected the forelimbs (FL), the hindlimbs (HL), the genital tubercle (GT), a piece of trunk corresponding to the lumbo-sacral region (T1); a piece of trunk corresponding to the sacro-caudal region (T2) and finally, a piece of trunk corresponding to the developing caudal region (T3, Fig 1C). As expected from the WISH experiments, *Hotair* transcripts were scored in the hindlimbs, the genital tubercle and the trunk samples T2 and T3. The highest steady-state levels of *Hotair* RNAs were detected in the GT and the T3 embryonic tissues (Fig 1D, S1 Dataset). As a control, *Hotair* transcripts were not detected in any tissues derived from homozygous *Del(Hotair)*^*-/-*^ mutant embryos (Fig 1D). To better compare this dataset with published results, we analyzed in parallel the RNA-seq data obtained from primary tail tip fibroblast (TTF), derived from both wild type and *Del(Hotair)*^*-/-*^ mice [20]. This analysis revealed that the expression level of *Hotair* in control TTF was very low when compared to its expression levels in the GT or the posterior T3 trunk sample (Fig 1E, S2 Dataset).

### Phenotype of mice lacking the *Hotair* lncRNA

*Hotair* was reported to be important for both the proper establishment of the mouse vertebral column and for the formation of the forelimb mesopodial articulation: the wrist [20,21]. To confirm this phenotypic effect, we inter-crossed *Del(Hotair)*^*+/-*^heterozygous mice and examined the skeletons of F1 animals at postnatal day 22 (P22). We investigated in particular the three reported sites of observed alterations in mutant *Del(Hotair)*^*-/-*^ mice [20,21]. We first searched for potential differences in vertebral formulae, as it was reported that 58% of *Del(Hotair)*^*-/-*^ mice had five lumbar vertebrae, while 100% of wild type CBA/C57/BL6 mice had six lumbar vertebrae [20].

**Fig 2:**
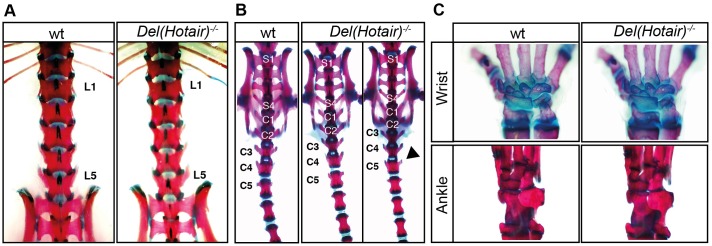
*Hotair* deletion has little impact, if any, on skeletal morphology. Alizarin Red and Alcian Blue skeletal staining of wild type and *Del(Hotair)*^*-/-*^ mice. (A) Lumbar region of wild type (left) and *Del(Hotair)*^*-/-*^ (right). In our (B6xCBA) background, both control and mutant animals have five lumbar vertebrae (L5), with an equally low incidence of L6 (see Table 1). (B) The sacro-caudal region of wild type (left) and *Del(Hotair)*^*-/-*^ (right) animals, with the black arrowhead pointing to a moderate gain of lateral protrusion in mutant caudal vertebra 5 (C5), usually not observed in control animals. (C) Normal wrist and ankle bones in both wild type (left) and *Del(Hotair)*^*-/-*^ (right) animals. The number and organization of mesopodial bones remained unchanged in the mutant condition.

All our mutant alleles at *Hox* loci (see e.g. [23]) are systematically backcrossed onto mixed (B6xCBA)F1 animals to maintain heterogeneous but similar backgrounds when comparing experimental crosses. After bringing the *Hotair* mutant mice [20] onto this genetic background for some generations, we found that 80% of *Del(Hotair)*^*-/-*^ mice displayed five lumbar vertebrae, similar to wild type littermates (Chi-square test, *p*-value 0.97, Fig 2A, Table 1). In both wild type and homozygous mutant animals, the L6 formula was sporadically scored, as well as the mixed L6/S1 vertebral type, often observed in our stocks. Despite a limited number of specimens observed, but together with the fact that we were unable to detect specific transcripts in the embryonic trunk at this vertebral level by two independent methods, we conclude that this lncRNA is very unlikely to have a function in the organization of this very flexible morphological boundary (see discussion).

**Table 1:** Phenotypic analysis of wild type and *Del(Hotair)*^*-/-*^ skeletons at post-natal day 22 (P22).

	wt	*Del(Hotair)*^*+/-*^	*Del(Hotair)*^*-/-*^
	(n = 11)	(n = 11)	(n = 10)
Lumbar vertebrae			
L6	1	1	1
L6/S1*	2	1	1
L5	8	9	8
Wrist			
normal	11	11	10
ill-formed	0	0	0
Ankle			
normal	11	11	10
ill-formed	0	0	0

* Mixed identity

Various control and mutant specimen were scored for having either five lumbar vertebrae (L5), six (L6) or a mixed L5-6/S1 vertebra. The mesopodial bones (wrist and ankle) were found normal in shape and number in all cases.

We next analyzed the morphology of caudal vertebrae in the post-sacral region. Previous analyses had concluded that mice with *Hotair* deletions had longer lateral processes on the fourth vertebra when compared to wild type animals, with full penetrance. In our case, we observed that three out of ten *Del(Hotair)*^*-/-*^ mutant mice had longer processes on the fifth caudal vertebra, compared to wild type (Fig 2B). This may indeed correspond to a very subtle morphological alteration in this region of the caudal spine, although the penetrance of this light phenotype is not 100%. Unlike the lumbo-sacral and wrist alterations [20], this particular tail vertebral morphology was also scored by Lai and colleagues when analyzing another mutant allele of *Hotair* [21].

Finally, and even though we were unable to detect any *Hotair* transcripts in the forelimbs of E12.5 mice embryos, unlike for hindlimbs (Fig 1B), we carefully examined both forelimb and hindlimb skeletons of wild type and *Del(Hotair)*^*-/-*^ mutant mice. We did not detect any alteration in limb morphology (Fig 2C and Table 1), in particular in the anatomy of the wrist, where malformations due to the loss of *Hotair* had been previously reported (Fig 2C). The same conclusion was reached concerning the hindlimbs, even though *Hotair* transcripts could clearly be scored in the proximal part. Altogether, we could not reproduce the reported phenotypic effects of *Hotair* deletion at two sites, the wrist and the lumbo-sacral region, where we were also unable to detect any *Hotair* transcripts. Regarding tail vertebrae, a slight effect could indeed be observed, poorly penetrant and likely dependent on the genetic background (see below).

### Transcription profiles of *Del(Hotair)*^*-/-*^ mutant embryonic tissues

**Fig 3:**
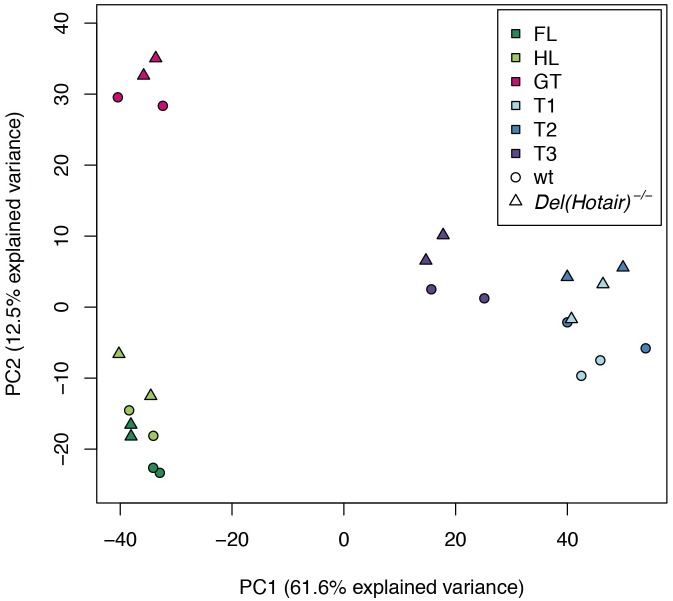
Overview of gene expression patterns in wild type and *Del(Hotair)*^*-/-*^ embryonic tissues. First factorial map for the principal component analysis (PCA) of gene expression levels. Tissues are color-coded (upper right corner) and the genotypes are indicated by either a circle (wild type) or a triangle (*Del(Hotair)*^*-/-*^). The numbers in parentheses indicate the proportion of the variance explained by PC1 or by PC2.

To more globally evaluate the effect of *Hotair* deletion upon developmental gene regulation, we performed a principal component analysis (PCA) using the expression levels of all autosomal protein-coding genes detected in our RNA-seq experiments (Materials and Methods). We observed a good separation of the data according to tissue type, although the T1 and T2 samples clustered together (Fig 3). Principal component 1 (PC1), which explained 61.6% of the total gene expression variance, separated the trunk segments (T1, T2 and T3) from the other embryonic tissues. Likewise, the differences between GT, HL and FL were resolved along PC2, which accounted for 12.5% of the total variance (Fig 3). Part of the variance was also explained by the genotypes and we observed that wild type and *Del(Hotair)*^*-/-*^ samples were separated along PC2 (Fig 3). Of note, the same separation between wild type and *Del(Hotair)*^*-/-*^ on PC2 was observed in all tissues, even in T1 and FL where *Hotair* is not expressed (Fig 3). In agreement with the results from the clustering between samples, we observed high expression level correlations among biological replicates for all tissues (S1 Fig). Furthermore, gene expression clustering based on pairwise Euclidean distances between samples (see Materials and Methods) showed a clear separation between four different groups: the limbs (FL and HL), the GT, the T3 trunk segment and the remaining T1 and T2 trunk segments (S2 Fig). Using this method, we observed a separation between genotypes only when the T3 sample was considered.

Altogether, these results point to the reproducibility of replicates and illustrate the good separation between tissues, with the exception of the T1 and T2 trunk samples. The high similarity in global gene expression between T1 and T2 likely reflects the spatial proximity of these two tissues, even though we cannot exclude some variation in the positioning of the precise T1/T2 boundary during dissection, which is a challenging task in such young embryos.

### Expression analysis of wild type *versus Del(Hotair)*^*-/-*^ embryonic tissues

Since *Hotair* was proposed to act as a repressor of gene expression in cultured fibroblasts [13,20], we conducted tissue-specific differential gene expression analyses between wild type and *Del(Hotair)*^*-/-*^ samples to assess such a potential function under physiological conditions. We only considered as significant an absolute expression fold change greater than 1.5 and we set the false discovery rate (FDR) threshold at 5%. By using these parameters, we observed between 64 and 588 protein-coding genes differentially expressed in the various tissues analyzed (S3 and S4 Datasets). We first compared all tissues that express *Hotair* in the wild type condition, i.e. the T2, T3, GT and HL samples, reasoning that potential differentially expressed *Hotair* targets should be identified in these contexts. However, we were not able to identify any common genes with altered expression between wild type and *Del(Hotair)*^*-/-*^ samples (S3 Fig), suggesting that the *Hotair* deletion does not affect the same set of genes in all tissues analyzed.

**Fig 4:**
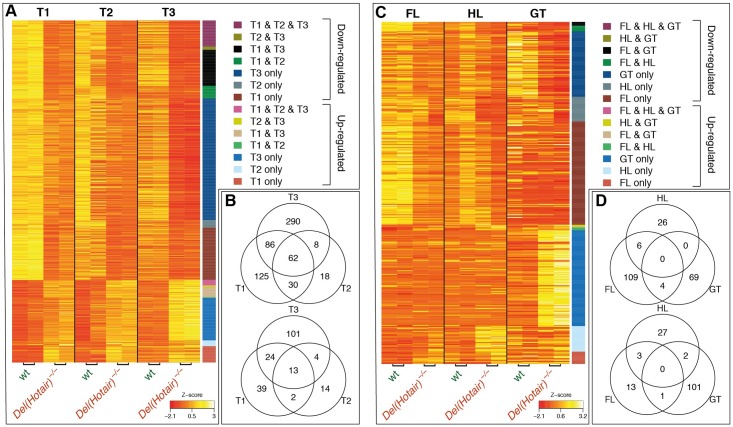
Differential expression between wild type and *Del(Hotair)*^*-/-*^ dissected samples. A-B) Differential gene expression analysis between wild type and *Del(Hotair)*^*-/-*^ trunk tissues (T1, T2, T3). The absolute fold change is > 1.5 and FDR < 0.05. The different columns correspond to sample type and rows correspond to differentially expressed genes. (A) Heat map of centered and scaled gene expression levels (Z-score log2 RPKM). Genes are color coded vertically, according to tissue and expression changes between genotypes. (B) Venn diagram showing the number of down-regulated (top) and up-regulated (bottom) genes. C-D) Differential gene expression analysis between wild type and *Del(Hotair)*^*-/-*^ forelimbs (FL), hindlimbs (HL) and genital tubercle (GT). The absolute fold change is > 1.5 and FDR < 0.05. (C) Heat map of centered and scaled gene expression levels (Z-score log2 RPKM). Genes are color coded vertically according to the tissue and orientation of expression between genotypes. (D) Venn diagram showing the number of down-regulated (top) and up regulated (bottom) genes.

We thus divided the differential expression analysis based on the global expression clustering results. First, we compared the trunk samples T1 (lacking *Hotair* expression), T2 and T3. We identified 62 down-regulated genes and 13 up-regulated genes between wild type and *Del(Hotair)*^*-/-*^ samples, which are shared in all trunk sections (Fig 4A and 4B). Of note, we observed a common trend in gene expression differences in all trunk samples, even though only some of them passed the established thresholds (Fig 4A). Gene ontology (GO) analysis for either common down-regulated or common up-regulated genes showed no enrichment of functional terms and the majority of differentially expressed genes were down-regulated, which was unexpected given the previously proposed role of *Hotair* as a repressor [20](Fig 4A and 4B). GO analysis of down-regulated genes in distinct *Del(Hotair)*^*-/-*^ trunk tissues revealed significant enrichment (FDR<10%) in functional terms related to organ development and multicellular organismal process for most tissues (S5 Dataset). Up-regulated genes in T3 were enriched for functional terms related to metabolic and biosynthetic processes and a weak enrichment for neuron differentiation genes was observed for T2 (S5 Dataset). Differential expression analysis for FL, HL and GT showed no common genes with altered expression (Fig 4C and 4D). GO analyses for differentially expressed genes in these tissues showed enrichment for functional terms related to development (S5 Dataset).

We next asked whether *Polycomb* target genes were preferentially up or down-regulated upon *Hotair* deletion. We defined putative target genes using H3K27me3 ChIP-seq data from wild type tail tip fibroblasts [20], selecting genes with a minimum 5-fold enrichment between H3K27me3 ChIP and input DNA in the gene promoter region (Materials and Methods) and thus obtained 861 putative target genes (S6 Dataset). We analyzed their pattern of differential expression in the T3 trunk sample, which includes the fetal tail and thus likely has the cell type composition *in vivo* most related to tail fibroblasts. Out of the 485 putative *Pc* targets that were expressed in the T3 segment (RPKM >1 in at least one wild type or *Del(Hotair)*^*-/-*^ sample), 60 genes were significantly differentially expressed (absolute fold change > 1.5 and FDR <10%), including 50 down-regulated and 10 up-regulated in the *Del(Hotair)*^*-/-*^ samples (S4 Fig). This indicates that only 17% of all differentially expressed *Pc* targets were up-regulated in *Del(Hotair)*^*-/-*^ samples, which is slightly lower than the proportion of up-regulated genes among non-targets (25% up-regulated genes out of 564 differentially expressed non-target genes, Chi-square test p-value 0.18). Thus, we could not detect any enrichment for up-regulation of putative *Pc* target genes when compared to all other expressed protein-coding genes. Therefore, under these physiological conditions, we could not find evidence supporting a role for *Hotair* in setting up, maintaining or re-enforcing the repression of this set of *Polycomb* target genes. While physiologically relevant, our analysis is however difficult to directly compare with the situation in tail fibroblasts, as *Polycomb* occupancy naturally depends on both the tissue-type and the developmental stage.

### Expression of imprinted genes in *Del(Hotair)*^*-/-*^ embryonic tissues

Interestingly, a subset of imprinted genes including *H19* and *Meg3* was shown to be up-regulated upon deletion of *Hotair* in TTF [20]. We thus analyzed the expression status of known imprinted genes transcribed (RPKM>1) in at least one sample (S7 Dataset). To ensure maximum sensitivity, we lowered our FDR threshold to 10% while maintaining an absolute expression fold change greater than 1.5. With these parameters, we observed a total of 21 imprinted genes differentially expressed in our samples (S5 Fig, S7 Dataset). We found that 71% of differentially expressed imprinted genes were down-regulated, while only 29% were up-regulated. Notably, *H19* and *Meg3* were down-regulated in our samples, in contrast to what was observed in TTF.

In conclusion, these global transcriptome analyses comparing *Hotair* deletion mutant and wild type micro-dissected tissues revealed changes in gene expression upon deletion of the *Hotair* locus. Noteworthy, we observed numerous expression changes not only in tissues that normally express *Hotair* at detectable levels, i.e. the T2, T3, GT and HL samples, but also in tissues like the anterior trunk (T1) or the forelimb (FL), where *Hotair* lncRNAs were not detected. This suggests that such observed differences in gene expression cannot be explained by a mere direct effect of the *Hotair* RNA. Potential explanations to these unexpected observations are discussed below.

### *Hox* genes expression in wild type and *Del(Hotair)*^*-/-*^ mutant mice

**Fig 5:**
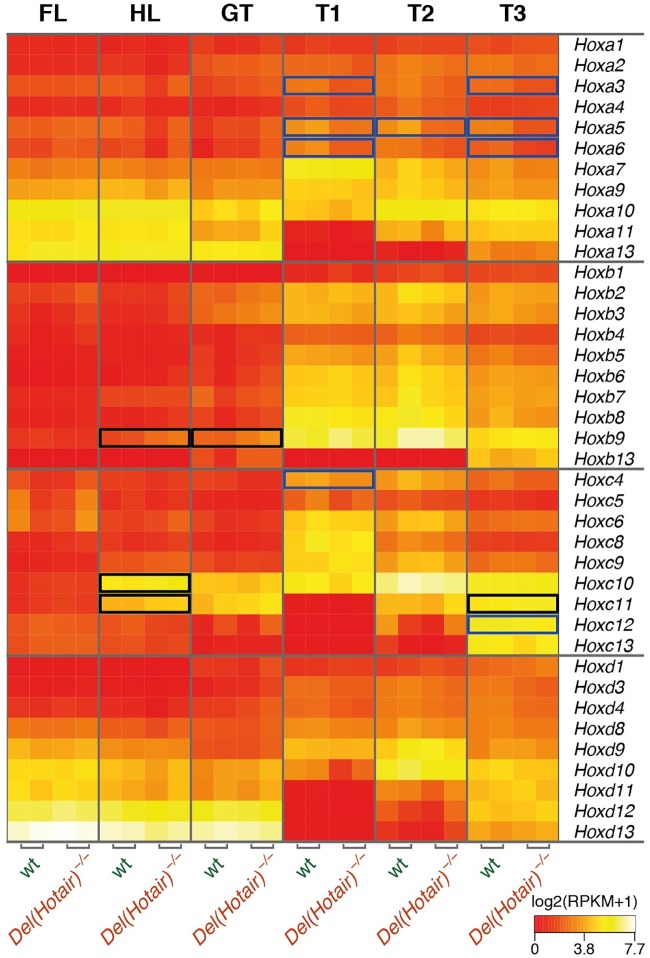
Expression of *Hox* genes in the various wild type and *Del(Hotair)*^*-/-*^ embryonic tissues. Heat map of log2-transformed RPKM expression levels for all *Hox* genes. The columns correspond to sample type (indicated on top) and the rows correspond to *Hox* genes (indicated on the right). The blue boxes point to down-regulated genes, whereas the black boxes indicate up-regulated genes (FDR < 10%, no minimal fold change threshold).

The original observation, which led *Hotair* to become the paradigm of lncRNAs acting in *trans*, was its capacity to regulate several genes members of the *HoxD* cluster by interacting with components of the *Polycomb* repressive complex 2 (PRC2)[13]. In contrast, no effect was initially reported upon *Hoxc* genes expression levels [13,20], despite the fact that *Hotair* is encoded from within the *HoxC* locus in both humans and mice [13,19]. We re-assessed this issue by analyzing the expression of all *Hox* genes across our various tissue samples (Fig 5). The global expression patterns of all four *Hox* clusters expectedly corresponded to previously described expression patterns for such embryonic stage and body levels (e.g. [24,25]).

In order to detect even subtle effects of *Hotair* upon *Hox* gene regulation, we lowered our FDR threshold to 10% for differential expression analyses. Under these conditions, we detected significant down-regulation of some anterior *Hoxa* genes (*Hoxa3*, *Hoxa5* and *Hoxa6*) in the three trunk samples (Fig 5 and S6 Fig). Interestingly, these differences were present in all trunk samples, including in T1 where *Hotair* is not expressed. We also observed a slight up-regulation of *Hoxb9* in HL and GT (Fig 5 and S6 Fig). Notably, in some of the tissues analyzed, we detected significant expression changes for both *Hoxc11* and *Hoxc12*, i.e. the two genes in the *HoxC* cluster that flank the *Hotair* locus (see below).

**Fig 6:**
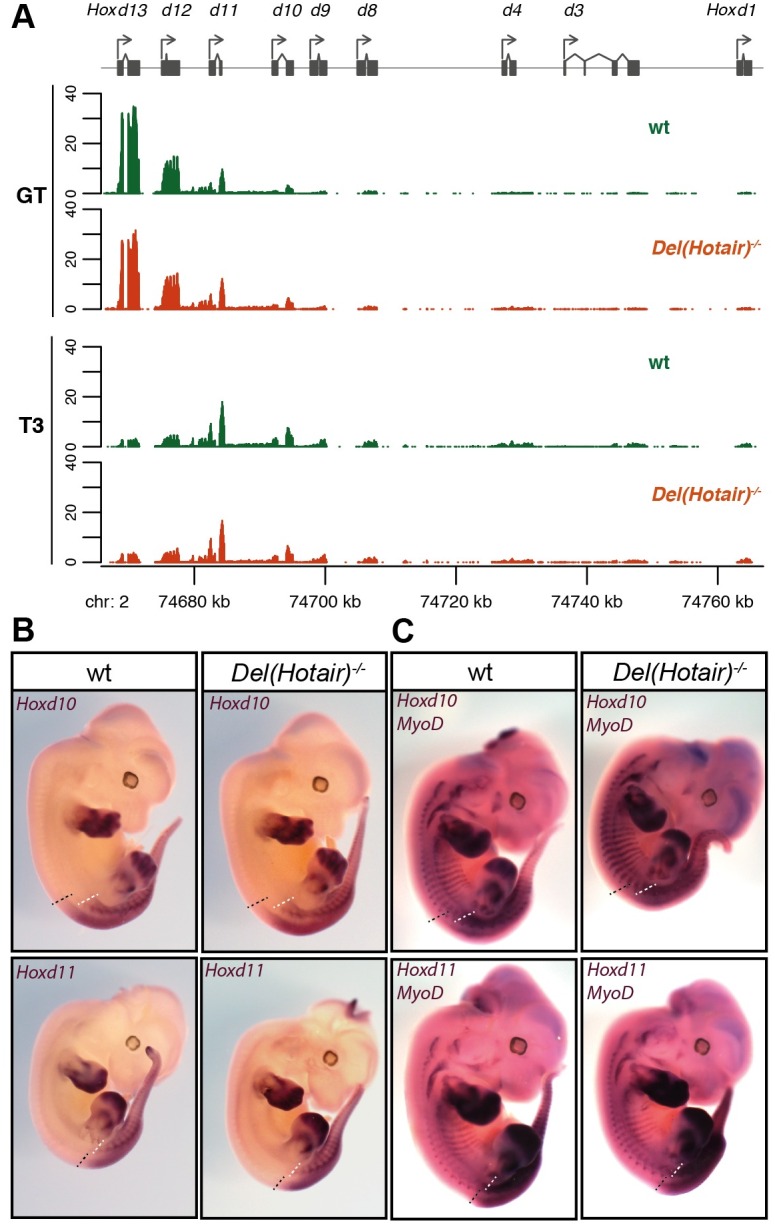
The deletion of *Hotair* does not alter *Hoxd* genes expression *in embryo*. (A) RNA-seq expression profiles of *Hoxd* genes in both the GT and T3 tissues of wild type (green) and *Del(Hotair)*^*-/-*^ (orange) E12.5 embryos. The Y-axis represents the per-base read coverage, normalized by dividing by the total number of million mapped reads in the corresponding samples. The two biological replicates were pooled for this representation and only uniquely mapping reads were used. (B) WISH of *Hoxd10* and *Hoxd11* on E12.5 wild type (left) and *Del(Hotair)*^*-/-*^ (right) embryos. The dashed lines indicate the rostral limits of the expression domains in the trunk, neural tube (black) and paraxial mesoderm (white). Adult vertebrae derive from the latter tissue. (C) Double WISH for the *MyoD* RNAs (for somite visualization) and either the *Hoxd10* (upper panel) or *Hoxd11* (lower panel) on E12.5 wild type (left) and *Del(Hotair)*^*-/-*^ (right) embryos. There was no detectable difference in the anterior limit of expression for any *Hoxd* gene analyzed.

However, in contrast with previous reports using tail fibroblasts [13,20], we did not detect any significant change in the steady-state levels of *Hoxd* genes RNAs, in any of the analyzed tissues (Figs 5 and 6A). To clarify this contradictory observation, we re-analyzed the previously published RNA-seq data from both wild type and *Del(Hotair)*^*-/-*^ TTF [20]. By implementing our analytical pipeline, we could not detect any significant difference in expression for any of the *Hoxd* genes (S7 Fig, S4 Dataset). Noteworthy, the expression levels of posterior *Hoxd* genes in this TTF dataset are either barely detectable or not detected at all, as for the *Hoxd12* gene, for example, suggesting that previous conclusions were raised based on particularly low transcript levels.

### Visualization of *Hoxd* genes expression in *Del(Hotair)*^*-/-*^ embryos

The deletion of *Hotair* was claimed to alter both the expression levels and the spatial transcript distribution of the *Hoxd10* and *Hoxd11* genes in the trunk [20]. We performed whole mount *in situ* hybridization (WISH) on both wild type and *Del(Hotair)*^*-/-*^ littermates to appreciate potential variations in the expression domains of these two genes. By using our well established protocol, we found that *Hoxd10* and *Hoxd11* transcripts showed wild type distributions in *Del(Hotair)*^*-/-*^ mutant specimen (Fig 6B). To more precisely evaluate any potential difference in these expression domains between homozygous mutant and control littermates, we carried out double WISH for the *Hox* gene of interest in combination with a probe specific for the *MyoD* gene, which allowed for unambiguous somite visualization [26]. In both wild type and *Del(Hotair)*^*-/-*^ embryos, *Hoxd10* was expressed in the future spine up to the level of somite 26, whereas *Hoxd11* was scored from somite level 29 and caudally, as previously reported [27]. Neither *Hoxd10*, nor *Hoxd11* showed any detectable increase in the intensity of the signal or in their spatial expression pattern, confirming the RNA-seq results (Fig 6A and 6B). Taken together, these observations suggest that *Hotair* has no effect on the regulation of *Hoxd* genes, at least in the developmental context and at the stage where the function of *Hox* genes is critical for morphological development.

### In-*cis* effect of *Hotair* deletion upon *Hoxc* genes transcription

**Fig 7:**
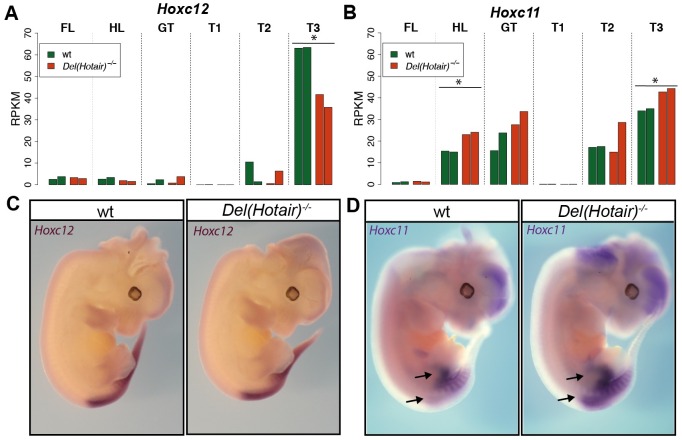
The deletion of *Hotair* affects the expression of the neighboring *Hoxc11* and *Hoxc12* genes. *Hoxc12* (A) and *Hoxc11* (B) expression (normalized RPKM values) in the various dissected tissue samples for wild type (green) and *Del(Hotair)*^*-/-*^ (orange) E12.5 embryos. The asterisk* indicates those samples where significant differences in transcript levels between genotypes were scored (FDR < 10%). (C) WISH using the *Hoxc12* probe in both wild type (left) and *Del(Hotair)*^*-/-*^ (right) E12.5 embryos. The spatial expression of *Hoxc12* remains globally unchanged. D) WISH of *Hoxc11* in wild type (left) and *Del(Hotair)*^*-/-*^ (right) E12.5 embryos. The arrows indicate the slight anterior shift in the expression profile and the increase in signal intensity in the mutant genotype.

Unlike for *Hoxd* genes, our differential expression analyses between *Del(Hotair)*^*-/-*^ and wild type samples revealed modest but significant changes for both *Hoxc11* and *Hoxc12*, the two genes neighboring the *Hotair* locus and thus flanking the deletion breakpoint (Fig 5). To further verify this new observation, we carefully analyzed the expression levels of both *Hoxc11* and *Hoxc12* in all tissue samples from where RNA-seq datasets had been obtained. As expected from their collinear transcription [28], *Hoxc12* transcripts were mainly detected in the most posterior T3 trunk sample. In this sample, a significant reduction in the level of *Hoxc12* RNAs was scored in *Del(Hotair)*^*-/-*^ specimen (Fig 7A). On the other hand, *Hoxc11* transcripts were detected in the hindlimbs (HL), the genital tubercle (GT) and the T2 and T3 trunk samples. In these tissues, we observed an up-regulation of *Hoxc11* RNAs upon deletion of the *Hotair locus*, which was statistically significant for both HL and T3 (Fig 7B). In addition, a strong positive correlation between the expression levels of *Hotair* and *Hoxc11* was detected in wild type samples (S8 Fig). The correlation was weaker between *Hotair* and *Hoxc12* expression (S8 Fig).

We asked whether these changes in expression of *Hoxc* genes in some *Del(Hotair)*^*-/-*^ samples were accompanied by alterations in their spatial expression patterns. We analyzed the expression of both *Hoxc12* and *Hoxc11* by WISH in *Del(Hotair)*^*-/-*^ E12.5 embryos and wild type littermates. We did not observe any change for *Hoxc12* expression, neither in the transcript domain, nor in the intensity of RNA signal (Fig 7C). In contrast, *Del(Hotair)*^*-/-*^ embryos showed a clear rostral expansion of the *Hoxc11* transcript domain in the trunk, as well as an increased signal intensity in both the hindlimb buds and the trunk, in agreement with our RNA-seq data (Fig 7D).

### The local impact of deleting the *Hotair* locus

**Fig 8:**
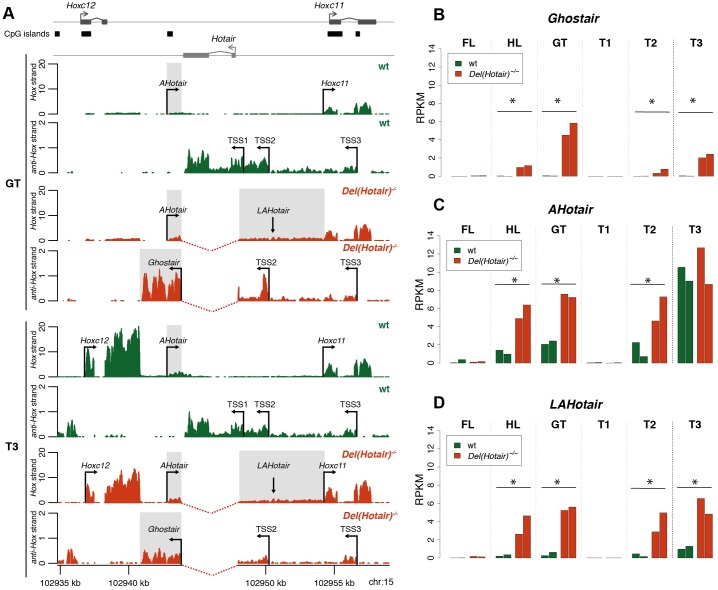
In-*cis* effects of the *Hotair* deletion on the local transcriptional activity. A) RNA-seq expression profiles of the genomic region neighboring *Hotair* in both the developing genitalia (GT, four profiles on top) and the most posterior trunk tissue sample (T3, four profiles at the bottom) from either wild type (green) and *Del(Hotair)*^*-/-*^ (orange) E12.5 embryos. In the wild type GT, only the *Hoxc11* gene is expressed along with *Hotair* on the opposite strand, which shows at least three putative start sites (arrows, TSS1 to TSS3). In the mutant GT, a long form of a new lncRNA (*AntiHotair*) now extends (grey box) on the *Hox* DNA strand, going over the deleted region up to the *Hoxc11* promoter. On the opposite DNA strand, the *Hotair* TSS2 and TSS3 are still functional and produce *Ghost of Hotair* (*Ghostair*), yet another new species of lncRNA, specific for the *Del(Hotair)*^*-/-*^ allele (in orange) and absent from the control allele (in green). A similar situation is observed in the T3 trunk sample, except that *Hoxc12* and *AHotair* are also expressed there. In the native locus, *anti-Hotair* is produced and meets with the end of the *Hotair* transcript. In the deleted allele, *Ghostair* is produced by the remaining *Hotair* TSS and terminates close to the 3’ end of the *Hoxc12* transcript (bottom two profiles). The gray boxes indicate the genomic regions used for the expression quantifications of *AHotair*, *LAHotair* and *Ghostair*. The Y-axis represents the per-base RNA-seq read coverage, normalized by dividing by the total number of million mapped reads in the corresponding samples. The two biological replicates were pooled for this representation and only uniquely mapping reads were used. B-D) Expression values (normalized RPKM) for *AHotair* (B), *LAHotair* (C) and *Ghost of Hotair* (D) in all tissue samples. Genotypes are color-coded with wild type in green and *Del(Hotair)*^*-/-*^ in orange. The asterisk* indicates those samples where significant differences in expression were scored between the two genotypes (FDR < 10%).

To understand more precisely the reason why the deletion of the *Hotair* locus impacted the transcription of the flanking *Hoxc* genes, we analyzed in details the RNA-seq profiles of the region comprising *Hoxc12*, *Hotair* and *Hoxc11*. We first asked if all transcript isoforms derived from the *Hotair* locus were abrogated in the *Del(Hotair)*^*-/-*^ allele and observed that the deleted region almost perfectly coincides with the annotated boundaries of the locus in the mouse. However, the inspection of the RNA-seq profiles in tissues that transcribe *Hotair* RNA revealed the existence of larger transcripts, extending over at least 2.4 kb upstream of the annotated promoter (Fig 8A). Although we cannot determine the precise location of *Hotair* transcription start site(s), the presence of continuous transcription upstream of the annotated gene boundaries indicates that at least one, and probably two of the possible *Hotair* promoters were not deleted. Indeed in *Del(Hotair)*^*-/-*^ tissues, we detected transcripts initiating upstream of the annotated *Hotair* promoter, for instance within the *Hoxc11* intron, and spanning over the deleted region (Fig 8A).

In contrast to its multiple start site(s), *Hotair* displayed a very sharp transcription termination site. In wild type tissues, transcription of *Hotair* terminated at the annotated site, with virtually no RNA-seq reads mapped downstream of this position (Fig 8A). However, in the *Del(Hotair)*^*-/-*^ samples, we observed transcription downstream of the deleted locus terminating within 100bp of the *Hoxc12* termination site (Fig 8A). The presence of this extended transcript, which likely derives from one of the native *Hotair* promoters (as predicted with a *de novo* transcript assembly procedure, S9 Fig), likely resulted from the deletion of the wild type *Hotair* termination signals (Fig 8A). To quantify this gain of transcription, we counted RNA-seq reads mapping on the region between the annotated *Hotair* termination site and the *Hoxc12* termination site, on the *Hotair* strand. We referred to this transcript, which only appears upon deletion of *Hotair*, as *Ghost of Hotair (Ghostair*). We observed significant gains of *Ghostair* transcription in *Del(Hotair)*^*-/-*^ samples, in all tissues that expressed *Hotair* in the wild type condition, i.e. the hindlimb buds, the genital bud and the two trunk samples T2 and T3 (Fig 8B).

### *Ghost of Hotair* and *Anti-Hotair*

Subsequent analyses of the RNA-seq profiles revealed an additional un-annotated promoter sequence, yet located on the *Hox* DNA strand. This promoter lies between *Hoxc12* and *Hoxc11* and overlaps with a CpG island (Fig 8A). In the wild type situation, it generates a relatively short, poorly abundant and un-spliced transcript, ca 1.8kb in size. The estimated termination site for this transcript was found within the region deleted in *Del(Hotair*), close to the termination of *Hotair* itself on the other strand. Accordingly, we refer to this short transcript as *Anti-Hotair* (*AHotair*). In *Del(Hotair)*^*-/-*^ samples, this CpG island promoter was still active, giving rise to a much longer *AHotair* transcript (Fig 8A, long *AHotair* or *LAHotair*), consistent with the deletion of its termination site. We did not observe any clear boundaries between this extended transcript and *Hoxc11*, suggesting that this *AHotair* RNA could leak onto the *Hoxc11* transcription unit. This was confirmed by a *de novo* transcript assembly procedure (see Materials and Methods, S9 Fig).

To quantify this gain of transcription from the *Hox* strand, we further defined two transcribed regions; The first one largely corresponded to the short *Anti-Hotair* transcript detected in wild type samples, starting at the CpG island promoter and ending at the boundary of the deleted region (Fig 8A). The second one, long *AHotair*, corresponded to the longer transcript observed in *Del(Hotair)*^*-/-*^ samples, starting at the deleted region boundary and ending at the annotated *Hoxc11* transcription start site (Fig 8A). In agreement with our observations based on the RNA-seq profiles, we detected significant increases in expression for both *AHotair* and *LAHotair* in *Del(Hotair)*^*-/-*^ mutant tissues (Fig 8C and 8D). Therefore, the deletion of the two annotated exons of *Hotair* [20] had a previously ignored important impact in *cis* by generating two new transcripts, which may potentially interact with the transcription of neighboring *Hoxc* genes.

## Discussion

### Homeotic *versus* Homeopathic phenotypes

In this study, we have re-investigated the phenotypic and molecular effects of deleting the *Hotair* lncRNA on mouse development *in vivo*, as reported in [20]. In this previous study, three phenotypic differences between wild type and *Del(Hotair)*^*-/-*^ mice were reported, namely wrist malformation, a posterior homeotic transformation from lumbar vertebra L6 to sacral vertebra S1 identity and a mild anterior homeotic transformation of the 4th caudal vertebra. We did not detect any wrist malformation, nor did we see any substantial homeotic phenotype in the lumbar region of mutant animals, thus contradicting two of the three reported phenotypic effects of the *Hotair* deletion. In *Mus musculus*, the lumbo-sacral transition shows great variability between L5 and L6 depending on the inbred strain considered and the total number of pre-sacral vertebrae. In fact, this number not only varies between inbred strains but also within the same strain and can even be biased by the sex of the animal [29]. Therefore, this region must be considered with great care before concluding on the presence of a homeotic transformation.

We note that another study involving two distinct deletion alleles of *Hotair*–though of smaller extents- also failed to confirm these latter two phenotypic effects [21]. The lack of effect of *Hotair* deletion upon wrist morphology is consistent with the absence of any detectable *Hotair* transcripts in mouse embryonic forelimbs (Fig 1) also reported previously [19] and by [21] using a sensitive *lacZ* reporter transgene system. Regarding the reported L5 to L6 transition, it is noteworthy that in wild type animals, detectable expression of *Hotair* in the paraxial mesoderm, i.e. in the mesodermal tissue that will generate the vertebrae, barely reaches the level of the lumbo-sacral transition, a transition labeled by its neighboring *Hoxc11* gene [30,31]. This makes a more anterior (at the L5 to L6 transition) *Hotair* loss-of-function dependent gain of function phenotype due to *Hoxd* genes difficult to understand.

Our analyses did nevertheless reveal a subtle difference between wild type and *Del(Hotair)*^*-/-*^ mice in the morphology of the post-sacral caudal vertebrae. Although this observation is in agreement with one of the previously described morphological alterations [20,21], we note that the penetrance of the mutant phenotype is much lower than the 100% reported by [20]. Also, such an anterior transformation should reflect a loss of function rather than the effect of de-repressed *Hox* genes [1,3], as already scored in some instances, for example when abrogating the function of the nearby located *Hoxc13* gene [32]. Moreover, we also observed variations in these vertebral morphologies amongst wild type animals. A potential explanation for the observed difference in phenotypic penetrance may reside in the genetic background of the animals. In this work, we used a mixed CBAxBL/6 strain, while previous studies used a BL/6 background. This relatively mild difference in genetic backgrounds may account, at least in part, for the discrepancy regarding the penetrance of this weak and physiologically poorly significant morphological transformation. Should this be the case, we would still have to conclude that the previously reported phenotypic effects of *Hotair* deletion are not only very mild but also inbred strain-specific, definitely arguing against a general role–even minor- of *Hotair* during mouse development. Accordingly, we would refer to these phenotypic alterations as homeopathic rather than homeotic [20].

### The effects of the *Hotair* deletion in *trans*

Using sensitive RNA-seq measurements, we showed that the expression of hundreds of genes changed significantly upon deletion of the *Hotair* locus *in vivo*. However, none of these changes in gene expression could be reconciled with the suggested role for *Hotair* in silencing gene expression *in vivo* [20]. In particular, the initial proposal that *Hotair* RNA acts in *trans* to repress the expression of posterior *Hoxd* genes and of a subset of imprinted genes *via* the recruitment of the PRC2 complex [13,20] was not supported by our results. Indeed we did not note any significant change either in the levels, or in the spatial distribution of *Hoxd* transcripts, in any of the tissues analyzed. Also, when a larger set of putative *Pc* target genes was considered, the same conclusion was reached (S4 Fig). Finally, the majority (71%) of the differentially expressed imprinted genes including the reported *Hotair* targets *H19* and *Meg3*, were down- rather than up-regulated in *Del(Hotair)*^*-/-*^ mutant samples, again in contradiction with previous results.

Therefore, our results are at odds not only with the phenotypic outcome of the *Hotair* deletion, but also with its effects upon gene expression [20]. One potential explanation to these serious discrepancies may be that the regulatory effect of *Hotair* is highly specific for tail tip or foreskin fibroblasts, which were previously used for functional investigations [13,20], whereas not at work *in vivo*, precisely in those embryonic tissues where *Hotair* is expressed at the highest levels. Indeed both *Hotair* and *Hoxd* genes transcripts are rather abundant in our tissue samples, while they are very weakly present in murine tail tip fibroblasts (Fig 1, S7 Fig). Unless *Hotair* would function more efficiently at low concentrations, we conclude that our *in vivo* system is better suited to reveal the role of *Hotair*, if any.

Another possibility is that the function of *Hotair* might not be exerted at the developmental stage analyzed (E12.5) but instead, at other time points. This explanation is nevertheless not compatible with the absence of phenotypic effects on skeletal morphology at P22, which should still be scored, should the deletion of *Hotair* deregulate target genes at other developmental stages. Also, the various genetic backgrounds may influence the penetrance of the phenotype (see above) and, by genotyping through the *Hotair* deleted locus, one may select for one particular haplotype associated with the mutant allele, which may result in some differential gene expression. Finally, it remains possible that a few hours difference in the developmental timing may lead to substantial relative variations in amounts of transcripts for many genes, in particular at an embryological stage where many important differentiation events occur.

In this context, it must be noted that the settings used for our transcriptome analyses overlap in sensitivity with the biological variations of the system itself, as seen for example with the variation in the level of *Hotair* in the GT replicate samples (Fig 1). Such differences can be due to intrinsic variations, yet most likely to slight variations in the micro-dissection plans or in the developmental stage of littermate embryos, or both. For example, a slight variation in the thickness of the piece in the trunk would elicit quantitative differences in *Hox* gene expression, whereas the depth of the piece (trunk) or the proximal level of the section (limbs, genitalia) may involve another presumptive tissue type, leading to large qualitative differences in transcripts. In fact, many of the strongest differentially expressed genes are clearly unrelated to those developmental processes involved in the potential morphological or molecular phenotypes under scrutiny (S10 Fig). This would also explain that differences are seen even in those samples where neither *Hotair*, nor *Hoxc11* are expressed. Accordingly, we do not interpret these results as reflecting changes in biological processes but, instead, as a sign of the sensitivity and intrinsic variations of our *in embryo* approach.

### The effects of the *Hotair* deletion in *cis*

When investigating the roles of lncRNAs by genetic approaches *in vivo*, it is often problematic to separate the lncRNA-dependent effects from those generated by the manipulation of the corresponding genomic locus [33]. *Hotair* is transcribed from within the *HoxC* cluster, a tightly packed and gene-dense locus, and its deletion was reported to have no consequence on the transcription of the neighboring *Hoxc* genes at the developmental stage and cell types examined [20]. Here again, our results *in embryo* contradict this view and showed that the expression levels of both *Hoxc11* and *Hoxc12* changed upon deleting the *Hotair* locus. We observed an extension in the spatial distribution of *Hoxc11* transcripts in both the trunk and the hindlimbs of *Del(Hotair)*^*-/-*^ mutant specimens. Upon examination of the datasets of [20], we also found differences for *Hoxc10* and *Hoxc12* between wild type and *Del(Hotair)*^*-/-*^ tail tip fibroblasts (S7 Fig). Therefore, the deletion of the *Hotair* locus had a significant impact in *cis* on *Hoxc* gene expression, in both *in vivo* and *in vitro* systems.

**Fig 9:**
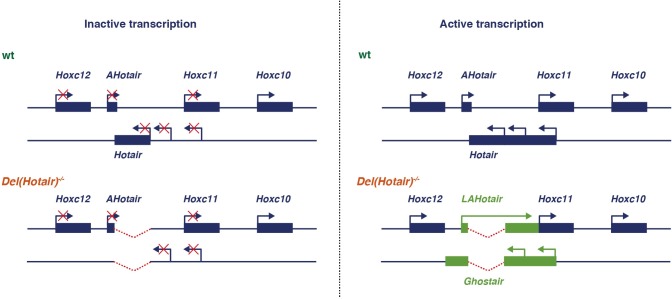
Schematic summarizing the data shown in Fig 8. In a wild type situation and at a body level anterior to *Hoxc11* expression (from example in the upper lumbar region such as L3 or L4), *Hoxc10* is active whereas the whole posterior part of the *HoxC* cluster is repressed (top left). Once *Hoxc11* and *Hoxc12* become activated, in more posterior regions of the body, both the *Hotair* and the *AntiHotair* RNAs are produced, from the anti-*Hox* and *Hox* DNA strands, respectively (top, right). Upon deletion of the *Hotair* locus, the posterior *HoxC* cluster remains closed for transcription in the anterior parts of the body (bottom, left). In contrast, the activation of the *Hoxc11* region (bottom right) triggers the transcription of *Ghostair* on the opposite DNA strand, which meets the 3’ end of the *Hoxc12* transcription unit, perhaps causing the light decrease in *Hoxc12* mRNAs. On the *Hox* strand, the anti-*Hotair* RNA can now cross over the deleted region and contribute to the transcription of *Hoxc11*, perhaps inducing the observed light gain of expression of the latter gene.

This was confirmed by the observation of *Ghost of Hotair* (*Ghostair*), a novel RNA produced by the anti-*Hox* strand in the deletion mutant allele. This transcript initiates at one of the alternative *Hotair* promoters, which was not included into the deletion, and terminates close to the 3’ end of the *Hoxc12* transcript on the opposite strand. Our analysis also revealed the existence of *AntiHotair*, a previously un-annotated transcript on the *Hox* strand, derived from a CpG island promoter located close to the 3’ end of *Hotair*. While in the wild type situation this transcript remains relatively short and ends within the region targeted by the *Hotair* deletion, a longer *AntiHotair* transcript was produced in *Del(Hotair)*^*-/-*^ samples with no clear separation with *Hoxc11*. As a consequence, this transcript could leak onto *Hoxc11*, acting as an alternative 5’ un-translated region, which gives a mechanistic basis for the light gain of *Hoxc11* expression in *Del(Hotair)*^*-/-*^ tissues (Fig 9). Such in-*cis* effects on the local transcription landscape by deleting transcription termination signals on both strands are likely independent from any possible *Hotair* function.

### *Hotair* back into context

The *Hotair* lncRNA is transcribed from within the *HoxC* gene cluster [13], i.e. one of the most gene-dense and GC-rich regions of mammalian genomes [34]. Due to the particular regulatory strategy at work on the four *Hox* gene clusters [8], any endogenous or exogenous promoter present within such a gene cluster will be transcribed at the place and time where the neighboring *Hox* genes will be activated. The transcription of *Hotair* is no exception to this rule, for transcripts are found posteriorly, roughly matching the expression domains of *Hoxc11* or *Hoxc12*. While it is indeed possible that *Hotair* exerts a genuine function during development, for example by micro-tuning the transcription of *Hoxc* genes in *cis*, the question as to whether or not this RNA could be a mere by-product of the complex regulation occurring in the gene cluster remains open, in our opinion.

In any case, a potential mis-regulation of *Hoxc* genes should be carefully considered when investigating *Hotair* functions. This is desirable not only when studying developmental phenotypes [18,19,35], where it may represent a confounding factor due to the known roles of *Hoxc* genes there, but also when studying the roles of *Hotair* in other biological processes including human diseases. For instance, it was reported that *Hotair* is overexpressed in breast cancer and that this RNA regulates metastasis by reprogramming chromatin *via Polycomb* complexes [36]. Our analysis of expression data obtained from a cohort of cancer samples [37] revealed a strong positive correlation between *Hotair* and *Hoxc11* expression (S8 Fig), also observed in our mouse wild type samples (S8 Fig). Therefore, while *Hotair* may indeed be involved in a variety of cancer conditions, it is likely that its over-expression in cancer cells is accompanied by *Hoxc11* over-expression, which may again confound the observed phenotypes.

### Conclusion

Thus far, four different alleles have been studied, which partially or entirely removed the *Hotair* lncRNA and no consensus has been found regarding a potential function of this RNA during mouse development [19–21]. In our hands, *Hotair* has no function during mouse development, *a fortiori* when the regulation of *Hoxd* genes in *trans* is concerned. The deletion of the locus engineered by [20] induces modifications in the transcription of some *Hoxc* genes, through complex re-allocations of promoter and termination sites leading to novel RNA species. This mis-regulation of *Hoxc* gene transcription may have a slight effect upon some vertebral morphologies, yet this impact–if any- would be poorly penetrant and inbred strain-specific, i.e. of little interest for our understanding of developmental processes at large. Yet another allele would be necessary to solve these discrepancies, whereby the CRISPR-cas9 technology would help abrogate the *Hotair* transcription without substantially modifying the in-*cis* environment. At this point however, we do not see the urgency of increasing the number of mutant alleles at this locus, as confounds due to genetic background differences may always blur the resolution of such subtle effects.

## Materials and Methods

### Mouse strains

The *Del(Hotair)* mouse strain was described in [20] and kindly provided by Dr. H. Chang. Heterozygous mice were crossed back onto a mixed CBAxC57/B6 background (Charles River). Wild type, heterozygous and homozygous mutant embryos were obtained by inter-crossing heterozygous mice. Genotyping was performed by PCR analysis on individual yolk sac lysates using the following primers:

Wild type (F)–CCTTATTCTCCCGGAGCCTAGC
Wild type (R)–CTGCCTCTGGCTCCACTCC
*Del(Hotair)-/-* (F)–CCTTGCCAACGTGTGGCTTCC
*Del(Hotair)-/-* (R)–CCAAGTCTACCGCTACACTGGC

### Ethics statement

Maintenance of, and experiments on animals were approved by the Geneva Canton ethical regulation authority (authorization GE/81/14 to D.D.) and performed according to Swiss law.

### Whole-mount *in situ* hybridization

Whole-mount *in situ* hybridizations (WISH) were performed according to standard protocols. Embryos were dissected in PBS and fixed from overnight in 4% paraformaldehyde (PFA), washed in PBS, dehydrated and stored in 100% methanol at –20°C. Both *Del(Hotair)*^*-/-*^ and control wild type E12.5 littermates embryos were processed in parallel to maintain identical conditions throughout the WISH procedure. DIG-labeled probes for *in situ* hybridizations were produced by *in vitro* transcription (Promega) and detection was carried out using an alkaline phosphatase conjugated anti-digoxigenin antibody (Roche). WISH probes templates were previously described in: *Hotair* [19]; *Hoxd10* and *Hoxd11 [27]*; *Hoxc11* [35]; *Hoxc12* [34]and *MyoD* [26].

### Skeletal Preparation

Whole mount skeletal preparation of P22 animals was done with standard Alcian blue/Alizarin red staining protocols.

### RNA extraction and RNA-seq library preparation

Embryonic tissues were stored at -80°C in RNAlater stabilization reagent (Ambion) before genotyping. After genotyping and embryo sorting, total RNA was extracted from tissues using QIAGEN RNeasy Plus Micro Kit after disruption and homogenization. RNA quality was assessed using an Agilent 2100 Bioanalyser. Only samples with high RNA integrity number were used. Sequencing libraries were prepared according to TruSeq Stranded mRNA Illumina protocol, with polyA selection. RNA-seq libraries were sequenced on an Illumina HiSeq 2500 sequencer, as single-end reads (read length 100 base pairs). We obtained between 36 and 54 millions of raw RNA-seq reads for each sample (S1 Table).

### RNA-seq processing and gene expression estimation

Raw RNA-seq reads were aligned on the mouse mm10 genome assembly using TopHat 2.0.9 [38]. Gene expression computations were performed using uniquely mapping reads extracted from TopHat alignments and genomic annotations from Ensembl release 82 [39]. We filtered the annotated transcript isoforms for protein-coding genes, keeping only transcripts annotated as ‘protein-coding’, thus discarding transcripts flagged as ‘retained_intron’, ‘nonsense-mediated decay’ etc. For *Hox* genes, we manually inspected annotated transcripts and retained only the canonical isoform for each gene, discarding read-through transcripts and retained introns. For non-coding genes, all annotated isoforms were kept. We then constructed ‘flattened’ gene models by combining the exon coordinates from all retained isoforms and counted the number of unique reads that aligned on these exons. We discarded reads that aligned on two or more overlapping genes on the same strand, as well as reads containing more than 2 mismatches or small insertions or deletions. We computed RPKM (Read per Kilobase of Exon per Million mapped reads) expression levels for each gene based on the unique read counts. The total number of mapped reads was computed on the entire nuclear genome, discarding reads that mapped on the mitochondria. RPKM expression levels were then further normalized across samples with a median scaling procedure, using as a standard the 100 genes with the least expression rank variation across samples, found in the 25%-75% range of expression levels [40]. As a control, we also computed expression levels using all TopHat mapped reads and the multi-read and fragment bias correction procedures implemented in Cufflinks [41]. The same procedure was applied for previously published tail tip fibroblast RNA-seq samples [20]. The RNA-seq data presented in this previous publication were also strand-specific and generated with a dUTP protocol that sequences the antisense mRNA strand like the TruSeq Stranded mRNA protocol.

### Statistical analyses and graphical representations

The principal component analysis (PCA) was performed using the dudi.pca function in the ade4 package in R [42]. The input table for the PCA consisted of log2-transformed RPKM expression levels, for all protein-coding genes that had RPKM >1 in at least one of our samples. The data was centered (meaning that the mean expression levels were brought to a value of 0 for each gene, removing between-gene variations in expression levels) but not scaled prior to the PCA analysis. Euclidean distances between samples were computed with the standard dist function in R and clustered using the hierarchical clustering method (hclust). All statistics and graphical representations were done in R.

### Differential expression analyses

We tested for differential gene expression using DESeq2 [43] in R. Specifically, we contrasted a generalized linear model that explains the variation in read counts for each gene, as a function of the genotype (wild type or *Del(Hotair)*^*-/-*^) with a null model that assumes no effect of the genotype. The analyses presented in the manuscript were performed with the likelihood ratio test (LRT); the Wald test was performed as a control and results are provided in the supplementary datasets. The tests were performed separately for each tissue. The p-values were corrected for multiple testing with the Benjamini-Hochberg approach, for all six tissues at the same time. The same procedure was applied for previously published tail tip fibroblast RNA-seq samples, which included wild type, heterozygous *Del(Hotair)*^*+/-*^ and homozygous *Del(Hotair)*^*-/-*^ samples. In this case, we performed three separate pairwise comparisons between the three genotypes.

### Gene ontology enrichments

We tested for gene ontology (GO) enrichment in the sets of differentially expressed genes using the GOrilla webserver [44]. Each enrichment analysis compared two lists of genes, the focal list containing differentially expressed protein-coding genes (up-regulated and down-regulated genes analyzed separately) and the background list containing all protein-coding genes expressed in the corresponding samples. To construct the background list, we computed the minimum number of reads observed for differentially expressed protein-coding genes, summed across all relevant samples and selected all genes that had equivalent or higher read counts.

### Prediction of *Polycomb* target genes

To obtain a list of putative *Polycomb* target genes, we analyzed chromatin immuno-precipitation followed by sequencing (ChIP-seq) data for H3K27me3 and corresponding input data, from wild type tail tip fibroblasts [20]. We mapped the ChIP-seq data on the mm10 mouse genome using Bowtie 2 [45]. We removed identical ChIP-seq reads to avoid biases stemming from PCR duplication and we kept unambiguously mapped reads with at most two mismatches. We computed the average H3K27me3 and input read coverage in the promoter region (defined as 2kb upstream the annotated transcription start site) for each Ensembl-annotated transcript. The same conclusions were reached when defining promoter regions as 4kb regions centered on the TSS (S6 Dataset). The read coverage was normalized by dividing by the total number of million mapped reads for each sample. We defined putative *Polycomb* targets as those genes for which the ratio between the H3K27me3 and input was at least 5, and for which the absolute H3K27me3 normalized coverage was at least 0.1. We discarded genes that had satellite repeats in the promoter regions as we observed that these repeats are enriched in H3K27me3 marks (likely as an artifact).

### Imprinted genes

The list of known mouse imprinted genes was extracted from http://www.geneimprint.com.

### *De novo* transcript assembly at the *HoxC* locus

We used RNA-seq data from our *Del(Hotair)*^*-/-*^ samples, combined across all six tissues, to predict transcript sequences for *Ghostair* and *AntiHotair*. To do this, we first split each RNA-seq reads into three segments and aligned them with Bowtie 2 on the DNA sequence delimited by *Hoxc12* and *Hoxc11*. We then extracted all RNA-seq reads that mapped at least partially onto this region and assembled transcripts *de novo* using Trinity [46] setting SS_lib_type = R since our data was strand-specific. We kept Trinity contigs with a minimum length of 1000bp and aligned them on the mouse chromosome 15 using BLAT [47]. We manually excluded small, repetitive BLAT hits. See also S8 Dataset.

### Expression data in cancer samples

To study the correlation between *Hotair* expression and the expression of neighboring genes *Hoxc11* and *Hoxc12*, we analyzed gene expression levels (RPM = reads per total million mapped reads) for a cohort of cancer samples [37].

## Supporting Information

S1 Fig Correlations between biological replicates. The scatterplots show the correlation of log2-transformed RPKM expression levels of all expressed protein coding genes between biological replicates (Pearson and Spearman correlation coefficients). FL, Forelimbs; HL, hindlimbs; GT, genital tubercle; T1, T2, T3; trunk samples corresponding to either the lumbo-sacral, the sacro-caudal region or the caudal region, respectively.
(TIF)

S2 Fig Pairwise Euclidean distances between samples. Hierarchical clustering and heat map of pairwise Euclidean distances between samples, computed on log2-transformed RPKM expression levels of all protein coding genes. The distances are color-coded, with blue representing small distances and yellow large distances.
(TIF)

S3 Fig Numbers of differentially expressed genes, in common among tissues. Venn diagrams of differential expression analysis results (fold change > 1.5 and FDR < 0.05) for all tissue samples that express *Hotair* in the wild type condition (T2, T3, GT and HL). (A) Venn diagram showing the down-regulated genes. (B) Venn diagram showing up-regulated genes.
(TIF)

S4 Fig Differential expression for candidate *Polycomb* target genes in the T3 trunk sample. (A) Volcano plot representing the log2 fold change and the false discovery rate (log10-transformed) for candidate *Polycomb* target genes (see Materials and Methods) in T3. The direction of expression changes is color-coded, with red showing down-regulated genes and green up-regulated genes. Non-significant genes are in blue. (B) Barplot representing the percentage of up-regulated and down-regulated genes for candidate *Polycomb* targets (left) and other genes (right).
(TIF)

S5 Fig Expression of known imprinted genes in wild type and *Del(Hotair)*^*-/-*^ embryonic tissues. Heat map of log2-transformed RPKM expression levels of all imprinted genes (extracted from http://www.geneimprint.com) expressed in our samples (RPKM>1, S7 Dataset). The columns correspond to samples and the rows correspond to imprinted genes. Blue boxes indicate down-regulated genes whereas black boxes indicate up-regulated genes (fold change > 1.5 and FDR < 10%).
(TIF)

S6 Fig Expression of *Hoxa* and *Hoxb* genes in wild type and *Del(Hotair)*^*-/-*^ dissected embryonic tissues. (A) RNA-seq expression profiles of the *HoxA* genomic region in the trunk T1 (top), T2 (middle) and T3 (bottom) samples of either wild type (green) or *Del(Hotair)*^*-/-*^ (orange) E12.5 embryos. Very subtle differences are scored (arrows), which are nevertheless considered as significant using our analytical parameters (see also Fig 5). The Y-axis represents the per-base RNA-seq read coverage, normalized by dividing by the total number of million mapped reads in the corresponding samples. The two biological replicates were pooled for this representation and only uniquely mapping reads were used. (B) RNA-seq expression profiles of the *HoxB* genomic region in the hindlimbs (HL, top) and genital tubercle (GT, bottom) of either wild type (green) or *Del(Hotair)*^*-/-*^ (red) E12.5 embryos. There again, the difference observed for *Hoxb9* (Fig 5) is weak yet considered as significant in our conditions (FDR < 10%, no fold change threshold).
(TIF)

S7 Fig Expression of *Hox* genes in tail tip fibroblasts (TTF). Bar plots showing the quantification of all *Hox* genes expression by RNA-seq (normalized RPKM values) in TTF. Datasets are from [20]. The FDR of the differential expression test (likelihood ratio test in DESeq2) between wild type and *Del(Hotair)*^*-/-*^ samples is indicated in red above each gene.
(TIF)

S8 Fig Correlations between the expression levels of *Hotair* and of the neighboring *Hoxc* genes. (A) Scatterplot of log2-transformed RPKM expression levels for *Hotair* and *Hoxc11* shows excellent correlation (R = 0.898). (B) Scatterplot of log2-transformed RPKM expression levels for *Hotair* and *Hoxc12* with a lower correlation coefficient (R = 0.592). The various tissues samples are represented by a color code and the genotypes are indicated by either a circle (wild type), or a triangle (*Del(Hotair)*^*-/-*^) (C) Scatterplot of log2-transformed RPM expression levels for *Hotair* and the *Hoxc11* gene in a cohort of 376 cancer samples [37], showing a high correlation coefficient (R = 0.92). (D) Scatterplot of log2-transformed RPM expression levels for *Hotair* and the *Hoxc12* gene in the same cohort as in C)[37], showing a lower correlation coefficient (R = 0.66). The Pearson correlation coefficients are shown above the plot.
(TIF)

S9 Fig *De novo* transcript assembly in the *HoxC* cluster for a pool of the various samples in *Del(Hotair)*^*-/-*^ mutant specimens. (A) Existing annotations for *Hoxc11*, *Hoxc12* and *Hotair*, as extracted from the Ensembl database. (B) Variation in the RNA-seq reads coverage at the vicinity of *Hotair*. The *Hox* and anti-*Hox* strands are depicted separately. The Y-axis represents the per-base RNA-seq read coverage, normalized by dividing by the total number of million mapped reads in the corresponding samples. All our *Del(Hotair)*^*-/-*^ samples were pooled for this representation and only uniquely mapping reads were used. (C) Genomic coordinates of *de novo* assembled transcripts, as predicted by Trinity on the basis of *Del(Hotair)*^*-/-*^ RNA-seq data and mapped on the genome with Blat (excluding repetitive hits). The different isoforms assigned to a single gene were combined for this representation. Note that transcripts may be fragmented, in particular at repeats and low complexity regions. We observe transcripts crossing the deleted region on both the *Hox* strand (Trinity identifier *TR5603|c0_g3*) and the anti-*Hox* strand (Trinity identifier *TR5645|c0_g1*). (D) Positions of repeated elements in the vicinity of *Hotair*.
(TIF)

S10 Fig Top differentially expressed genes in embryonic tissue samples. The volcano plots show the log2 fold change and the false discovery rate (log10-transformed) for differentially expressed genes (fold change > 1.5 and FDR < 0.05) in the various tissue samples. The top differentially expressed genes are highlighted in red (fold change > 4 and FDR < 0.00001). Dashed lines mark absolute fold changes of 2 and 4. FL, Forelimbs; HL, hindlimbs; GT, genital tubercle; T1, T2, T3; trunk samples corresponding to either the lumbo-sacral, the sacro-caudal region or the caudal region, respectively.
(TIF)

S1 Dataset Gene expression data for wild type and *Del(Hotair)*^*-/-*^ embryonic mouse tissue samples. Data are from forelimb buds (FL), hindlimb buds (HL), genital tubercle (GT) and the trunk T1, T2 and T3 samples. Numbers of uniquely mapped reads and RPKM/FPKM values are provided in separate files.
(TGZ)

S2 Dataset Gene expression for either wild type, *Del(Hotair)*^*+/-*^ or *Del(Hotair)*^*-/-*^ tail tip fibroblasts (TTF), after re-analysis of the RNA-seq datasets from Li and colleagues [20]. Numbers of uniquely mapped reads and RPKM/FPKM values are provided in separate files.
(TGZ)

S3 Dataset Differential expression analyses comparing wild type and *Del(Hotair)*^*-/-*^ mouse tissue samples. Data are from the FL, HL, GT, T1, T2 and T3 and are provided in separate files.
(TGZ)

S4 Dataset Differential expression analyses comparing wild type, *Del(Hotair)*^*+/-*^ and *Del(Hotair)*^*-/-*^ tail tip fibroblasts (TTF), after our re-analysis of the RNA-seq data from [20]. (TGZ)

S5 Dataset Gene ontology (GO) enrichment analysis, contrasting genes that are differentially expressed between wild type and *Del(Hotair)*^*-/-*^ mouse tissues from a background set of genes expressed at comparable levels in the same tissues. The enrichment analysis was done for the GO category ‘biological process’, using the GORILLA webserver [44].
(TGZ)

S6 Dataset Analysis of putative *Polycomb* target genes, predicted on the basis of H3K27me3 ChIPSeq data from Li and colleagues [20]. The table provides the H3K27me3 and input read coverage for the predicted *Polycomb* targets, as well as differential expression results for these genes in the T3 trunk segment.
(TGZ)

S7 Dataset Results of the differential expression analyses for imprinted genes. The list of imprinted genes was extracted from www.geneimprint.com.
(TGZ)

S8 Dataset Results of the *de novo* transcript assembly procedure, which we used to confirm that *Ghostair* and *AntiHotair* cross the deleted region. (TGZ)

S1 Table Basic statistics for the RNA-seq dataset, including the number of raw and mapped RNA-seq reads and the number of detected genes for each sample. (XLSX)
