# Supplementary figures and images for: Hotair Is Dispensible for Mouse Development
Source: PLoS Genet. 2016 Dec 15;12(12):e1006232. doi: 10.1371/journal.pgen.1006232 (PMC5157951; doi:10.1371/journal.pgen.1006232)

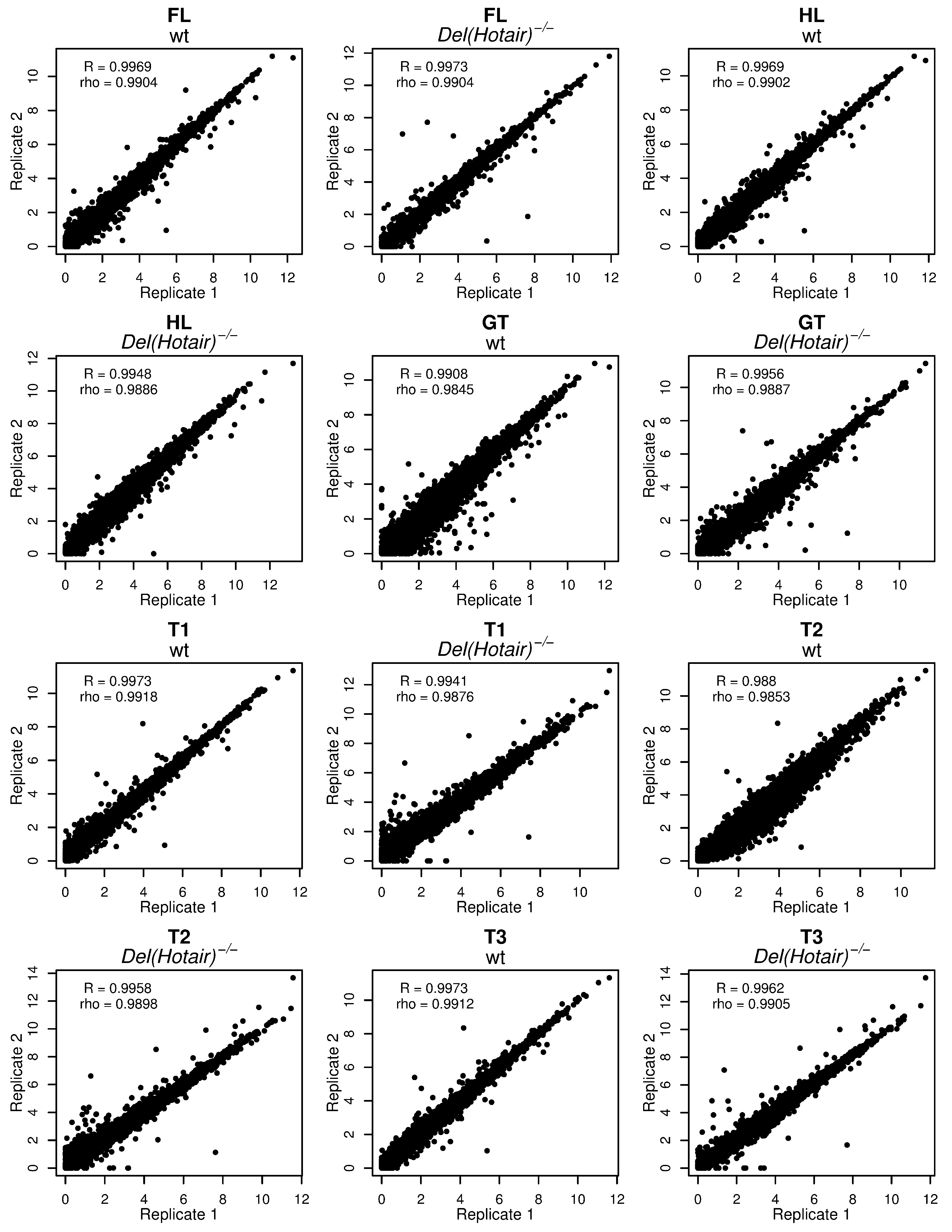

Supplement: S1 Fig — The scatterplots show the correlation of log2-transformed RPKM expression levels of all expressed protein coding genes between biological replicates (Pearson and Spearman correlation coefficients). FL, Forelimbs; HL, hindlimbs; GT, genital tubercle; T1, T2, T3; trunk samples corresponding to either the lumbo-sacral, the sacro-caudal region or the caudal region, respectively. (TIF) [file pgen.1006232.s001.tif]

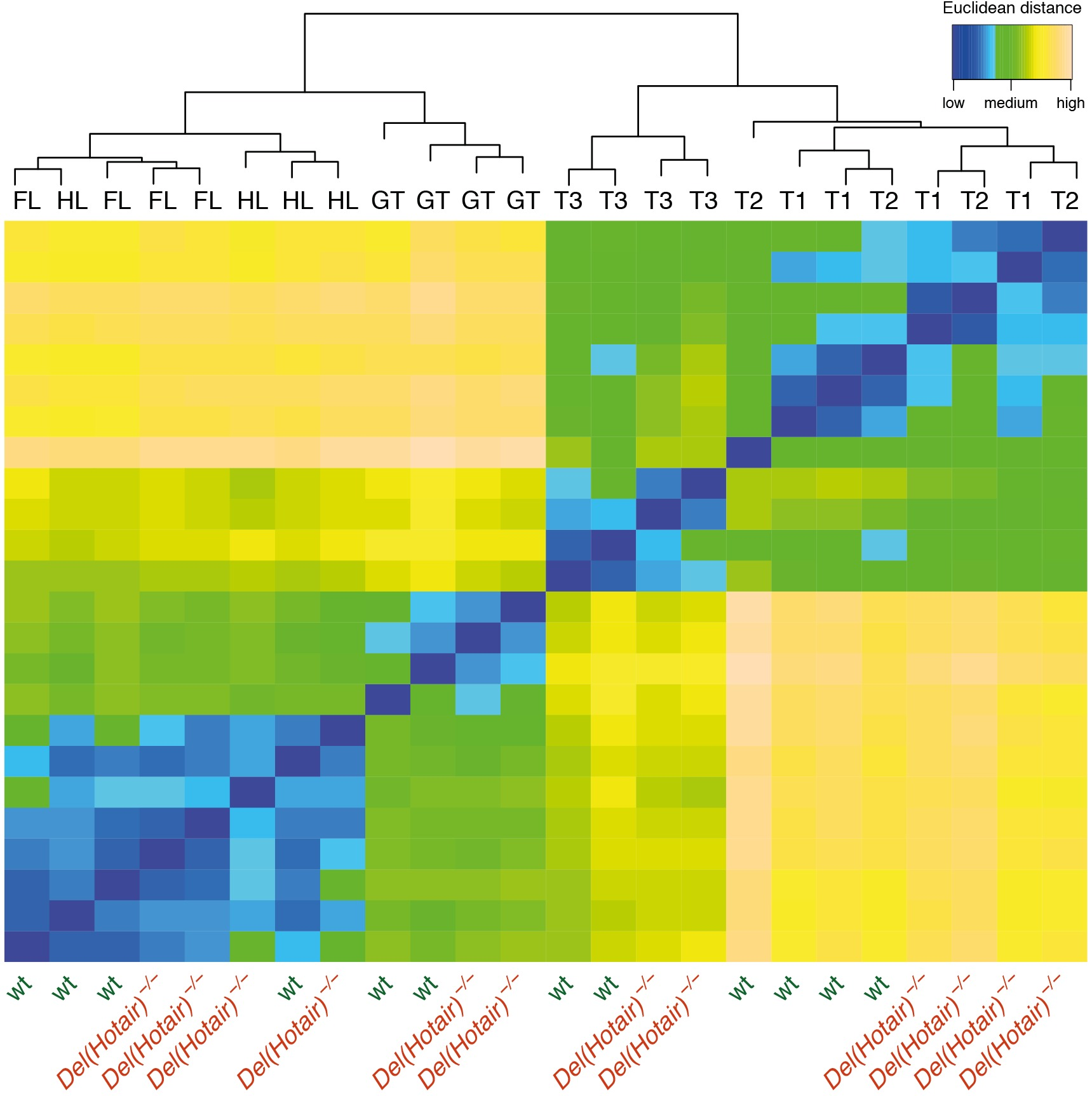

Supplement: S2 Fig — Hierarchical clustering and heat map of pairwise Euclidean distances between samples, computed on log2-transformed RPKM expression levels of all protein coding genes. The distances are color-coded, with blue representing small distances and yellow large distances. (TIF) [file pgen.1006232.s002.tif]

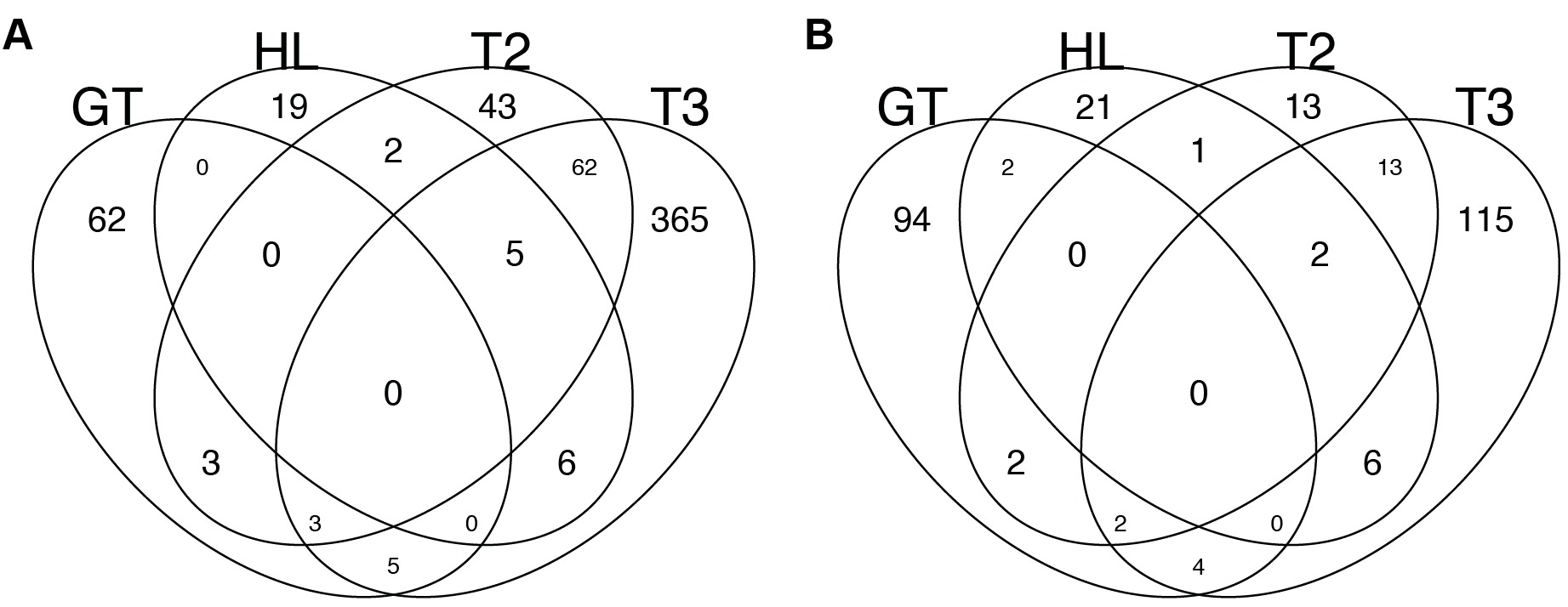

Supplement: S3 Fig — Venn diagrams of differential expression analysis results (fold change > 1.5 and FDR < 0.05) for all tissue samples that express Hotair in the wild type condition (T2, T3, GT and HL). (A) Venn diagram showing the down-regulated genes. (B) Venn diagram showing up-regulated genes. (TIF) [file pgen.1006232.s003.tif]

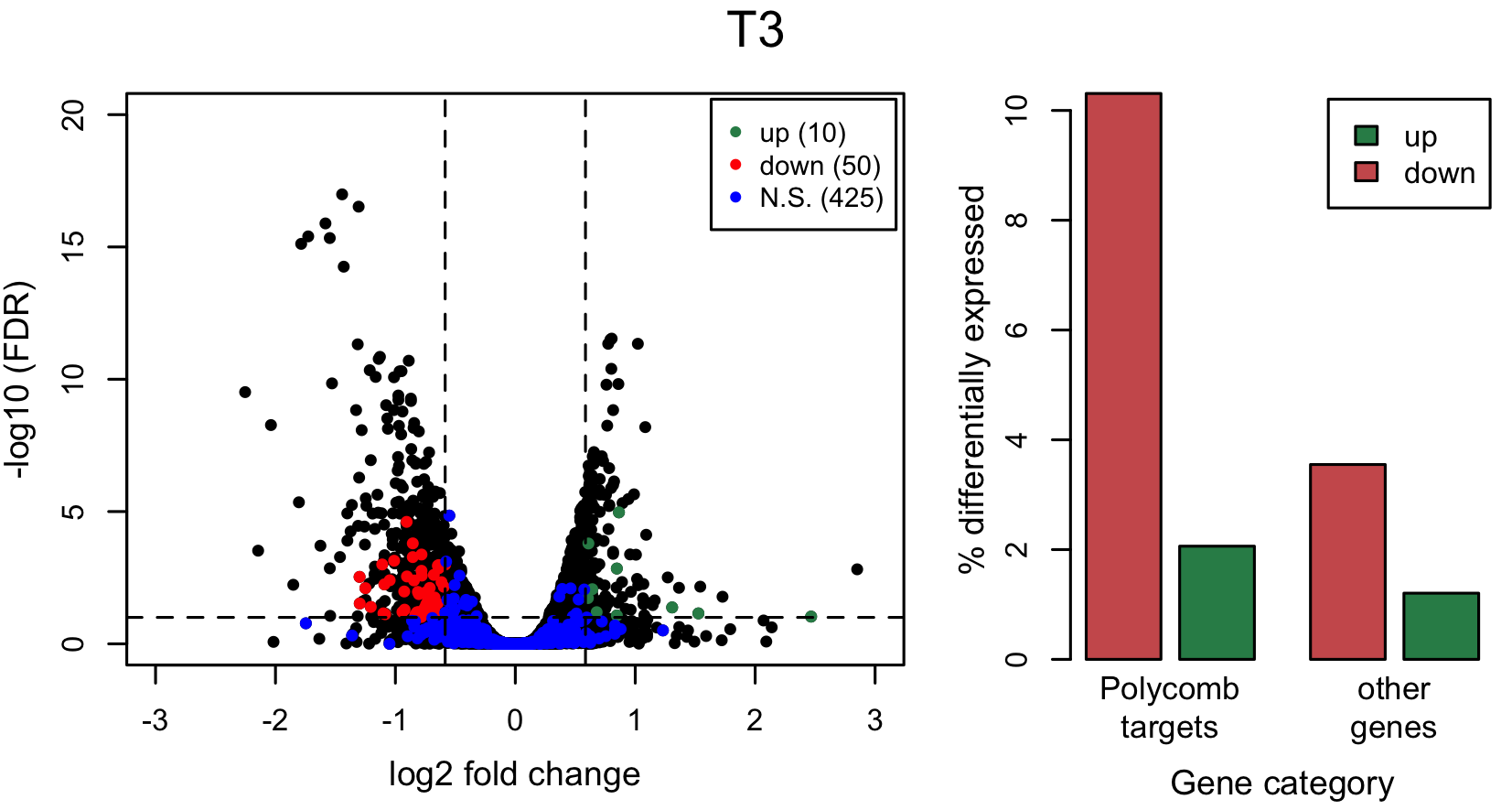

Supplement: S4 Fig — (A) Volcano plot representing the log2 fold change and the false discovery rate (log10-transformed) for candidate Polycomb target genes (see Materials and Methods) in T3. The direction of expression changes is color-coded, with red showing down-regulated genes and green up-regulated genes. Non-significant genes are in blue. (B) Barplot representing the percentage of up-regulated and down-regulated genes for candidate Polycomb targets (left) and other genes (right). (TIF) [file pgen.1006232.s004.tif]

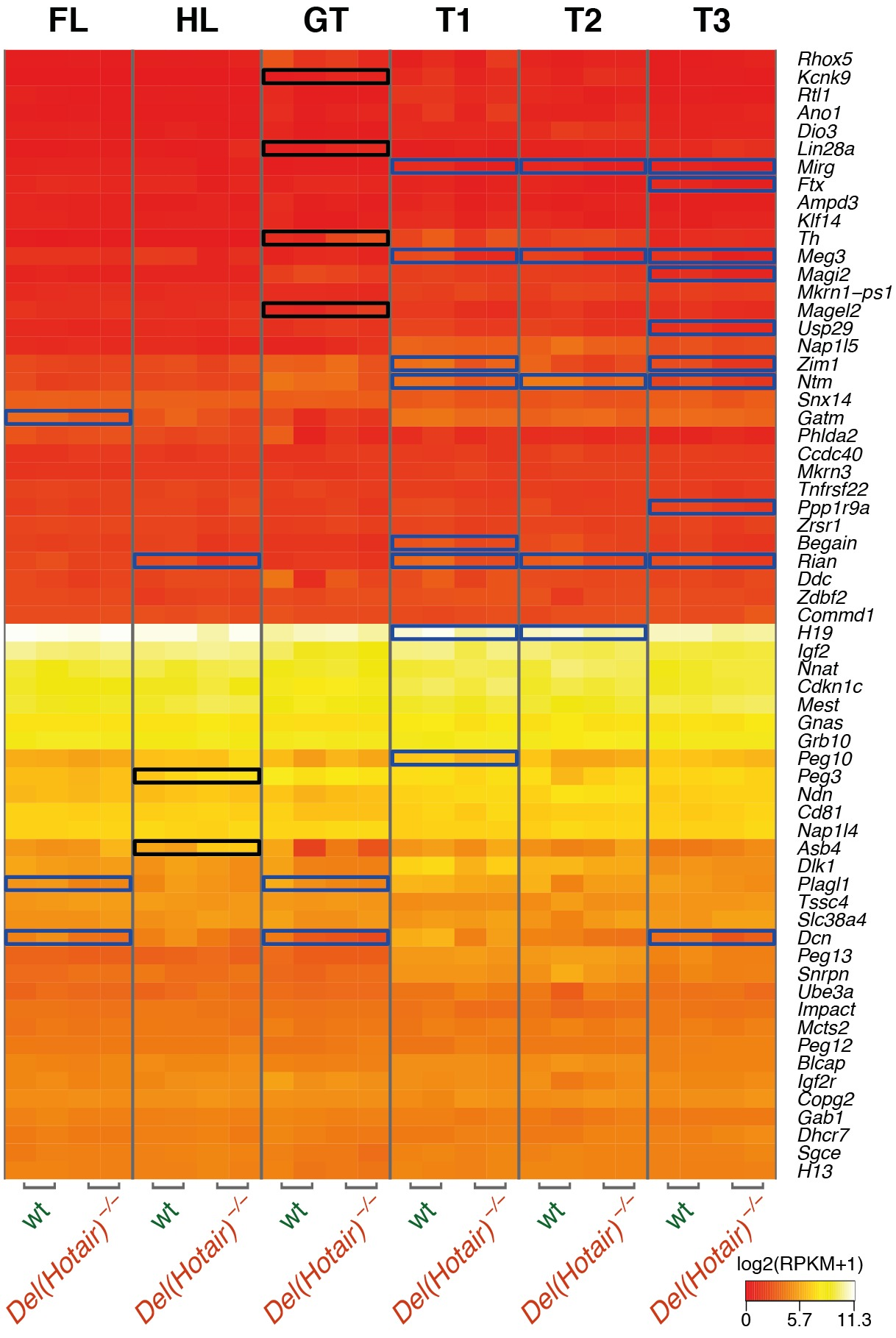

Supplement: S5 Fig — Heat map of log2-transformed RPKM expression levels of all imprinted genes (extracted from http://www.geneimprint.com) expressed in our samples (RPKM>1, S7 Dataset). The columns correspond to samples and the rows correspond to imprinted genes. Blue boxes indicate down-regulated genes whereas black boxes indicate up-regulated genes (fold change > 1.5 and FDR < 10%). (TIF) [file pgen.1006232.s005.tif]

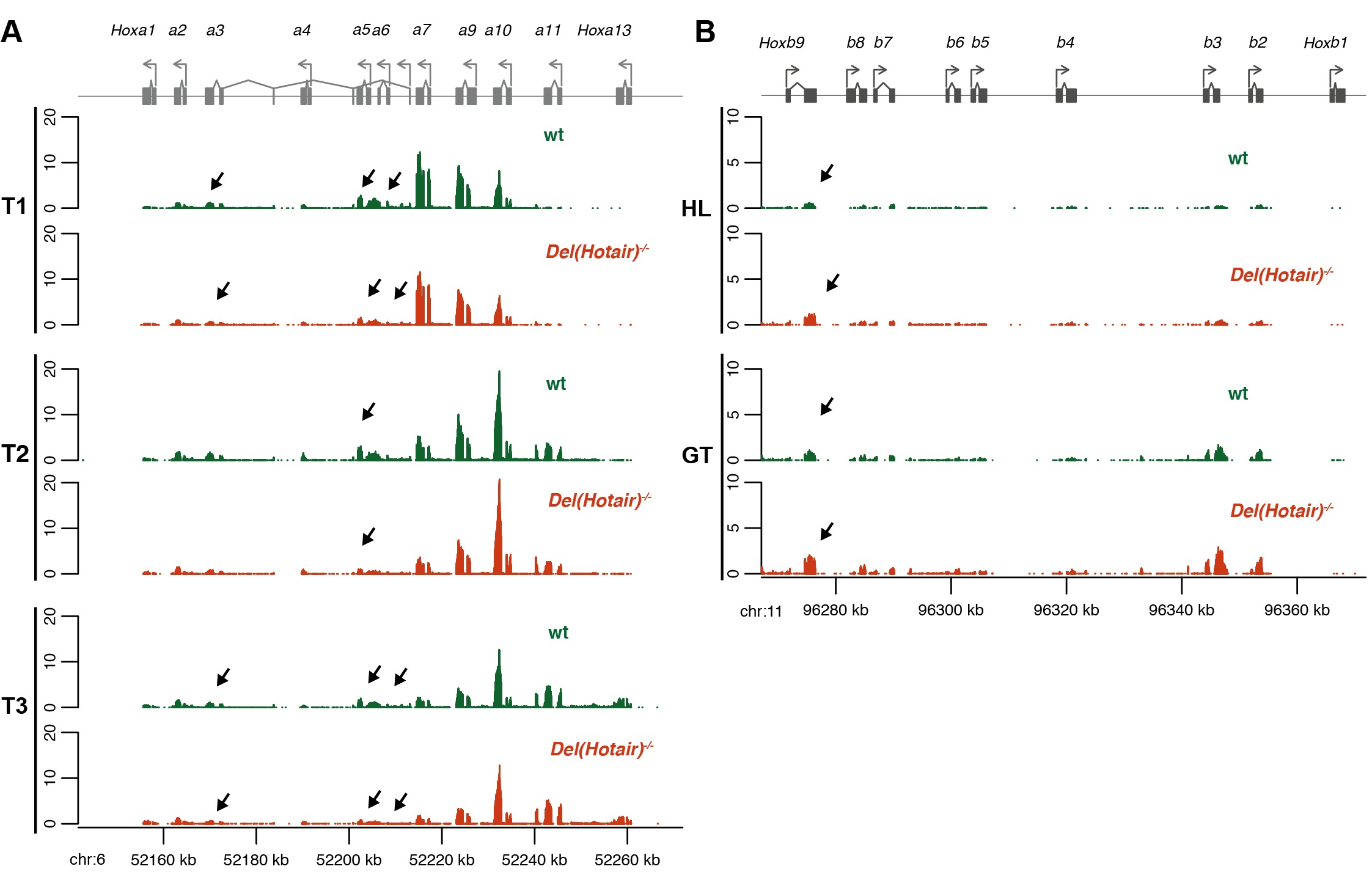

Supplement: S6 Fig — (A) RNA-seq expression profiles of the HoxA genomic region in the trunk T1 (top), T2 (middle) and T3 (bottom) samples of either wild type (green) or Del(Hotair)-/- (orange) E12.5 embryos. Very subtle differences are scored (arrows), which are nevertheless considered as significant using our analytical parameters (see also Fig 5). The Y-axis represents the per-base RNA-seq read coverage, normalized by dividing by the total number of million mapped reads in the corresponding samples. The two biological replicates were pooled for this representation and only uniquely mapping reads were used. (B) RNA-seq expression profiles of the HoxB genomic region in the hindlimbs (HL, top) and genital tubercle (GT, bottom) of either wild type (green) or Del(Hotair)-/- (red) E12.5 embryos. There again, the difference observed for Hoxb9 (Fig 5) is weak yet considered as significant in our conditions (FDR < 10%, no fold change threshold). (TIF) [file pgen.1006232.s006.tif]

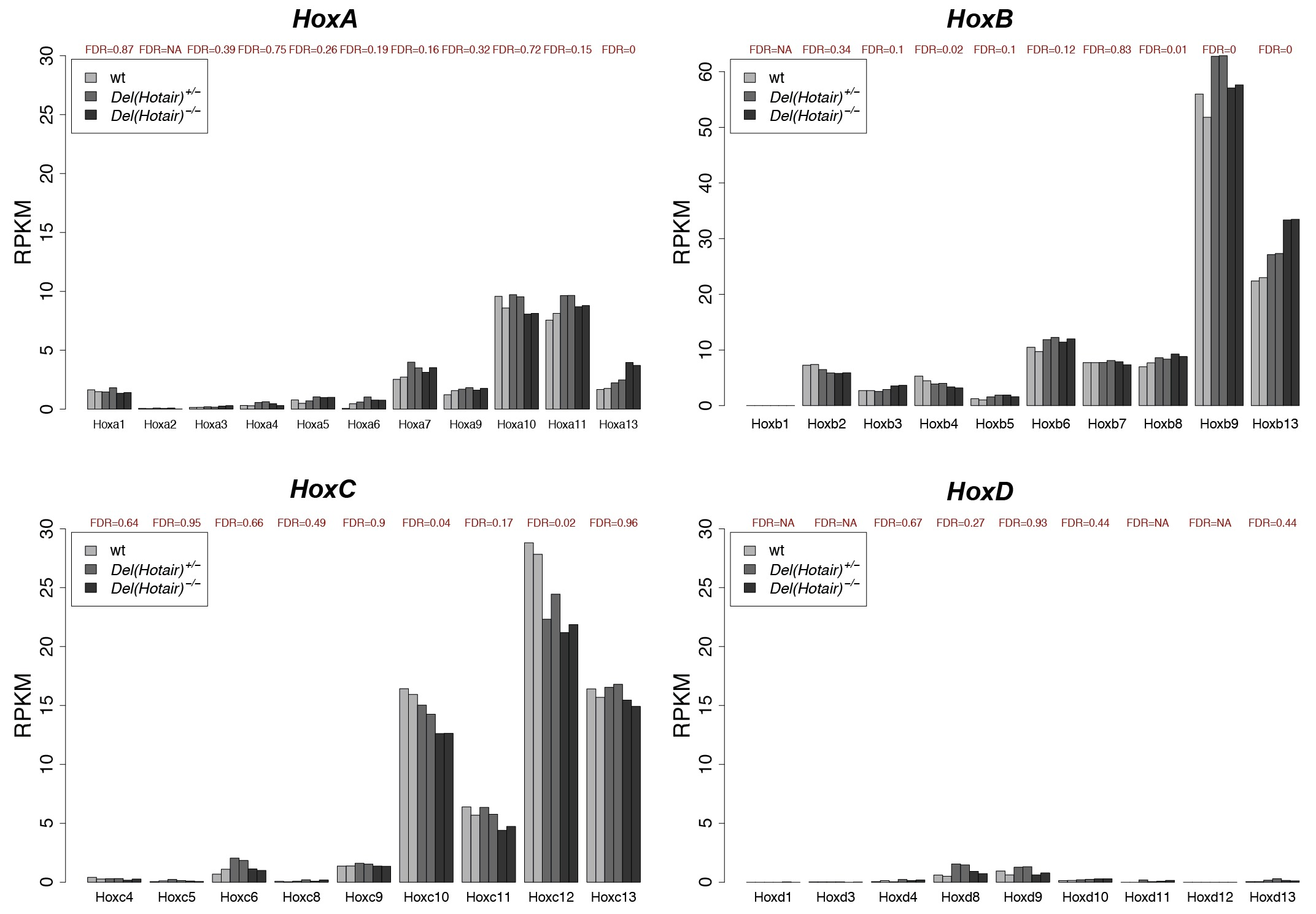

Supplement: S7 Fig — Bar plots showing the quantification of all Hox genes expression by RNA-seq (normalized RPKM values) in TTF. Datasets are from [20]. The FDR of the differential expression test (likelihood ratio test in DESeq2) between wild type and Del(Hotair)-/- samples is indicated in red above each gene. (TIF) [file pgen.1006232.s007.tif]

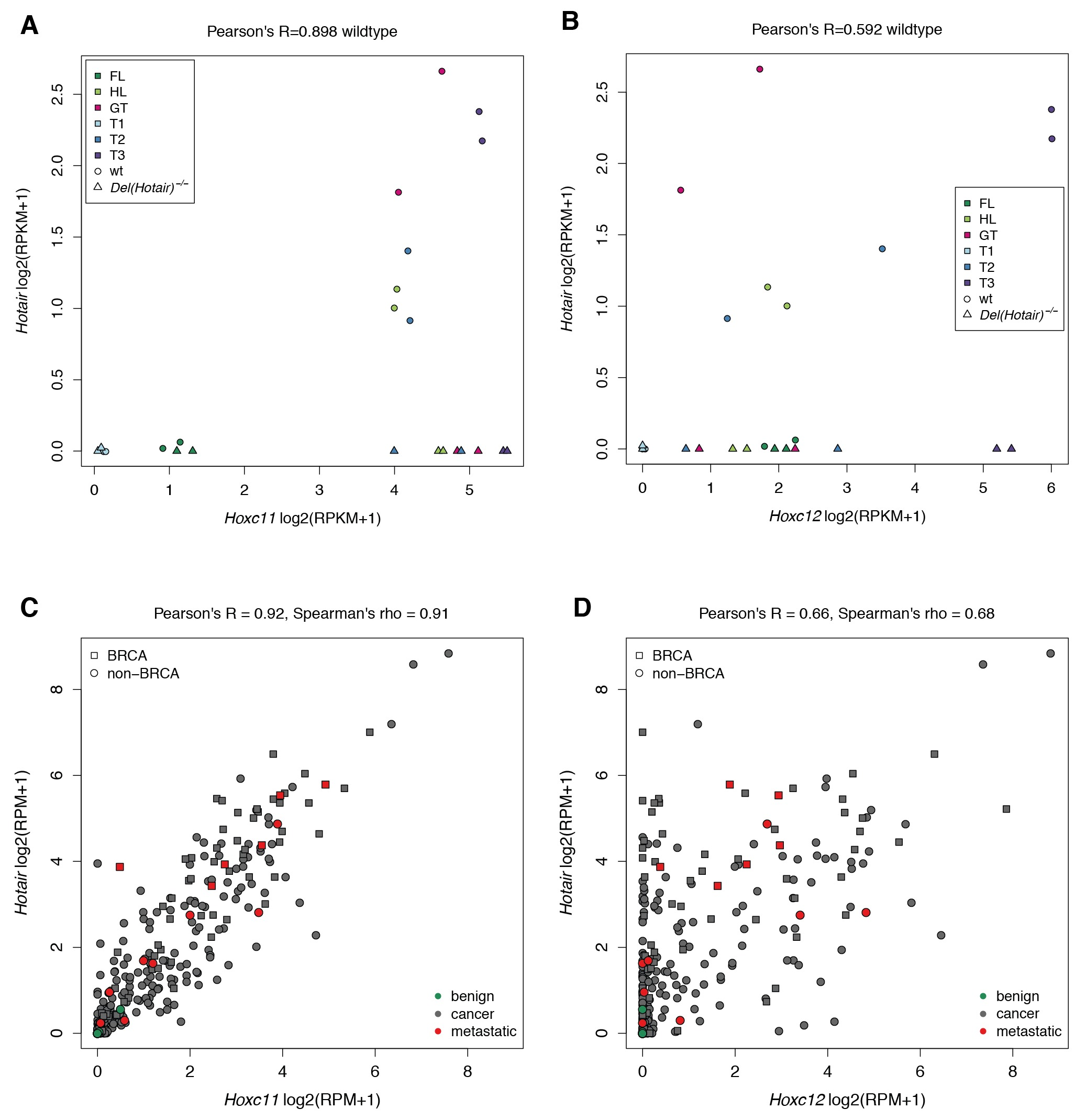

Supplement: S8 Fig — (A) Scatterplot of log2-transformed RPKM expression levels for Hotair and Hoxc11 shows excellent correlation (R = 0.898). (B) Scatterplot of log2-transformed RPKM expression levels for Hotair and Hoxc12 with a lower correlation coefficient (R = 0.592). The various tissues samples are represented by a color code and the genotypes are indicated by either a circle (wild type), or a triangle (Del(Hotair)-/-) (C) Scatterplot of log2-transformed RPM expression levels for Hotair and the Hoxc11 gene in a cohort of 376 cancer samples [37], showing a high correlation coefficient (R = 0.92). (D) Scatterplot of log2-transformed RPM expression levels for Hotair and the Hoxc12 gene in the same cohort as in C)[37], showing a lower correlation coefficient (R = 0.66). The Pearson correlation coefficients are shown above the plot. (TIF) [file pgen.1006232.s008.tif]

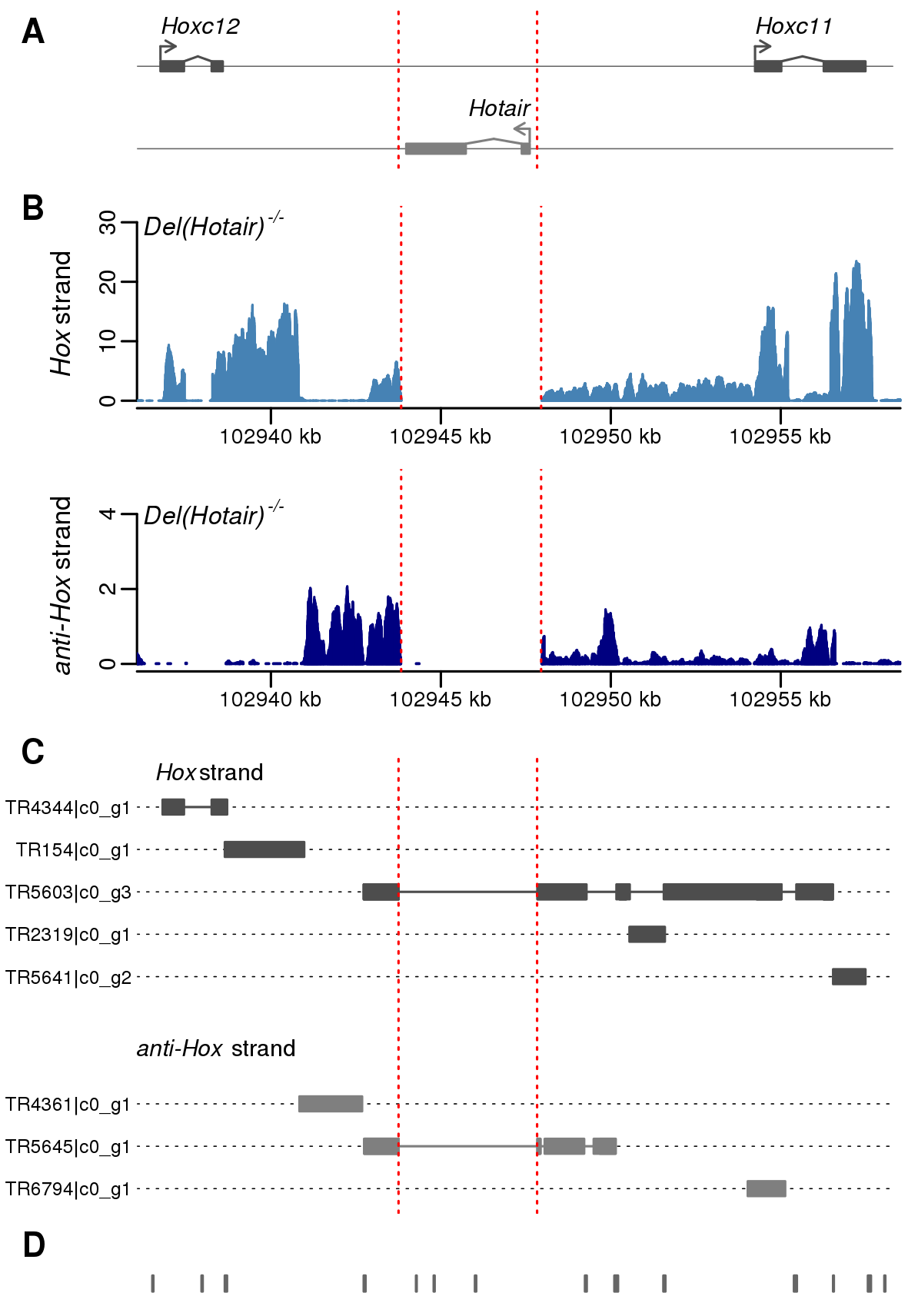

Supplement: S9 Fig — (A) Existing annotations for Hoxc11, Hoxc12 and Hotair, as extracted from the Ensembl database. (B) Variation in the RNA-seq reads coverage at the vicinity of Hotair. The Hox and anti-Hox strands are depicted separately. The Y-axis represents the per-base RNA-seq read coverage, normalized by dividing by the total number of million mapped reads in the corresponding samples. All our Del(Hotair)-/- samples were pooled for this representation and only uniquely mapping reads were used. (C) Genomic coordinates of de novo assembled transcripts, as predicted by Trinity on the basis of Del(Hotair)-/- RNA-seq data and mapped on the genome with Blat (excluding repetitive hits). The different isoforms assigned to a single gene were combined for this representation. Note that transcripts may be fragmented, in particular at repeats and low complexity regions. We observe transcripts crossing the deleted region on both the Hox strand (Trinity identifier TR5603|c0_g3) and the anti-Hox strand (Trinity identifier TR5645|c0_g1). (D) Positions of repeated elements in the vicinity of Hotair. (TIF) [file pgen.1006232.s009.tif]

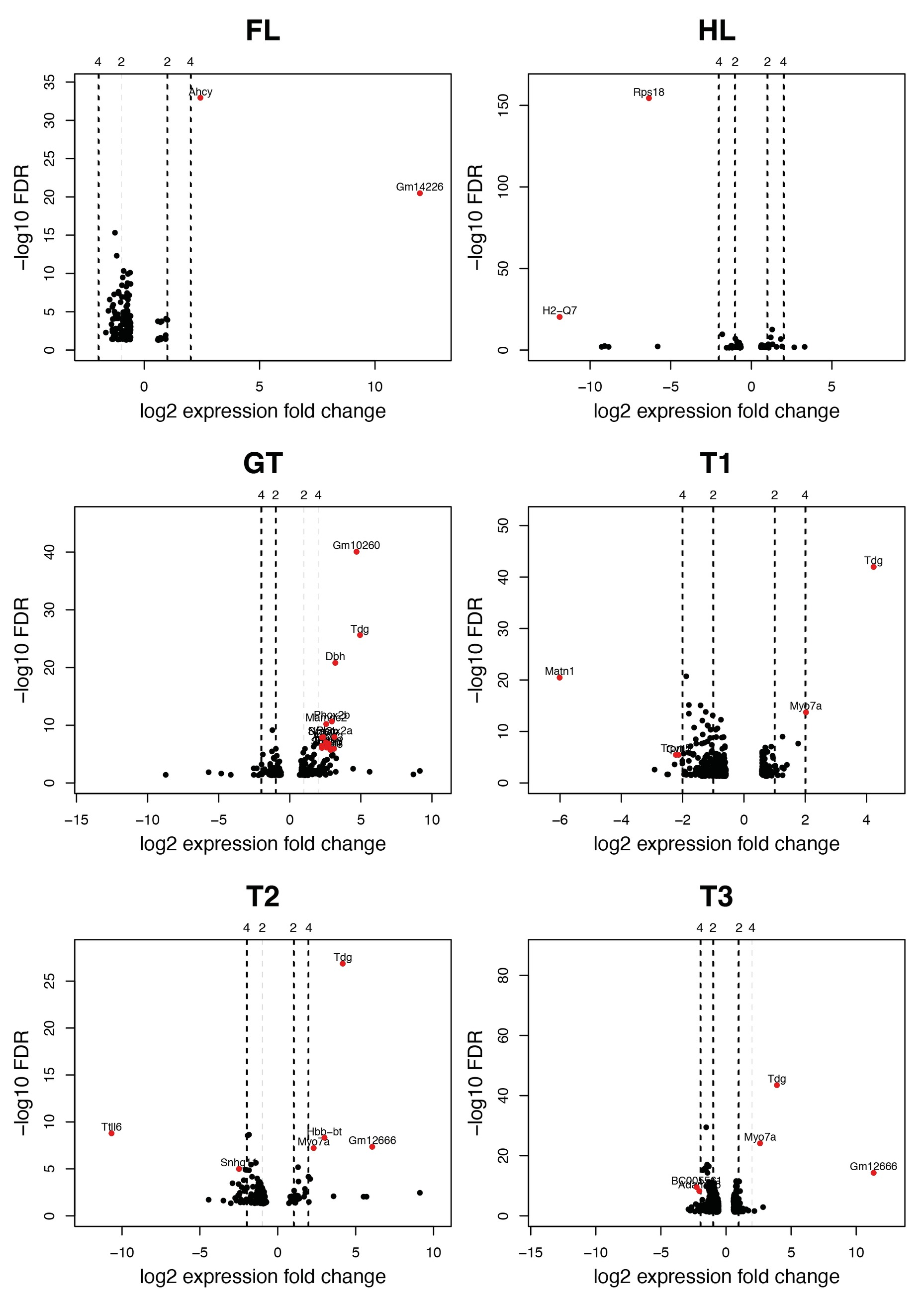

Supplement: S10 Fig — The volcano plots show the log2 fold change and the false discovery rate (log10-transformed) for differentially expressed genes (fold change > 1.5 and FDR < 0.05) in the various tissue samples. The top differentially expressed genes are highlighted in red (fold change > 4 and FDR < 0.00001). Dashed lines mark absolute fold changes of 2 and 4. FL, Forelimbs; HL, hindlimbs; GT, genital tubercle; T1, T2, T3; trunk samples corresponding to either the lumbo-sacral, the sacro-caudal region or the caudal region, respectively. (TIF) [file pgen.1006232.s010.tif]
